# The Combined Toxic Effects of Polystyrene Microplastics and Arsenate on Lettuce Under Hydroponic Conditions

**DOI:** 10.3390/toxics13020086

**Published:** 2025-01-24

**Authors:** Li Mu, Ziwei Gao, Mengyuan Wang, Xin Tang, Xiangang Hu

**Affiliations:** 1 Tianjin Key Laboratory of Agro-Environment and Safe-Product, Key Laboratory for Environmental Factors Control of Agro-Product Quality Safety (Ministry of Agriculture and Rural Affairs), Institute of Agro-Environmental Protection, Ministry of Agriculture and Rural Affairs, Tianjin 300191, China; gaoziwei6688@163.com (Z.G.); tengweimeng@163.com (M.W.);; 2 Key Laboratory of Pollution Processes and Environmental Criteria (Ministry of Education), Tianjin Key Laboratory of Environmental Remediation and Pollution Control, College of Environmental Science and Engineering, Nankai University, Tianjin 300350, China; huxiangang@nankai.edu.cn

**Keywords:** arsenate, defense mechanisms, metabolic pathways, transport, plant development, polystyrene microplastics

## Abstract

The combined pollution of microplastics (MPs) and arsenic (As) has gradually been recognized as a global environmental problem, which calls for detailed investigation of the synergistic toxic effects of MPs and As on plants and their mechanisms. Therefore, the interaction between polystyrene microplastics (PS-MPs) and arsenate (AsO_4_^3−^) (in the following text, it is abbreviated as As(V)) and its toxic effects on lettuce were investigated in this study. Firstly, chemisorption was identified as the main mechanism between PS-MPs and As(V) by the analysis of adsorption kinetics, adsorption thermodynamics, and Fourier transform infrared spectroscopy (FTIR). At the same time, the addition of As(V) promoted the penetration of PS-MPs through the continuous endodermal region of the Casparis strip. Furthermore, compared with the CK group, it was found that the co-addition of As(V) exacerbated the lowering effect of PS-MPs on the pH value of the rhizosphere environment and the inhibitory effect on root growth. In the P20V10 group, the pH decreased by 33.0%. Compared to the CK group, P20, P20V1, and P20V10 decreased the chlorophyll content by 68.45% (16 SPAD units), 71.37% (17.73 SPAD units), and 61.74% (15.36 SPAD units) and the root length by 19.31% (4.18 cm), 50.72% (10.98 cm), and 47.90% (10.37 cm) in lettuce. P5V10 and P20V10 increased CAT content by 153.54% (33.22 U·(mgprol)^−1^) and 182.68% ((38.2 U·(mgprol)^−1^)), Ca by 31.27% and 37.68%, and Zn by 41.85% and 41.85%, but the presence of As(V) reduced Na by 22.85% (P5V1) and 49.95% (P5V10). The co-exposure significantly affected the physiological and biochemical indicators as well as the nutritional quality of the lettuce. Finally, the metabolomic analysis of the lettuce leaves showed that combined pollution with PS-MPs and As(V) affected the metabolic pathways of the tricarboxylic acid cycle (TCA cycle), sulfur metabolism, and pyruvate metabolism. This study provides data for pollution management measures for co-exposure to PS-MPs and As(V).

## 1. Introduction

Microplastics (MPs), defined as plastic particles with a diameter of less than 5 mm, represent a newly recognized class of pollutants. In recent years, many studies have been conducted on co-contamination with MPs and other pollutants and their ecological safety and environmental hazards, but it remains an underexplored area. Further research is needed to gain a deeper understanding of the issue. Research has indicated that MPs have the potential to enhance the bioavailability of heavy metals [1]. Electron microscopy of MP particles revealed that their surface has many wrinkles and porous structures [2]. These features endow the particles with a large specific surface area, pronounced hydrophobic properties, and enhanced durability [3]. MPs exhibit an inherent ability to adsorb pollutants. They are capable of interacting with heavy metal contaminants present in the environment [4]. Upon the adsorption of metal ions and oxides by MPs, there is a subsequent transformation in their particle size, surface properties, and electrical characteristics. These changes subsequently modify the interaction patterns of MPs with metal ions and their potential toxicity to environmental organisms. Furthermore, MPs with particle sizes of 0.02 and 0.2 μm have been shown to enhance the migration of goethite and hematite within a quartz sand matrix, while these minerals also encourage the adsorption of 0.2 and 2 μm MPs onto the quartz sand surface [5]. At a pH level of 4, both polystyrene microplastics (PS-MPs) and quartz sand surfaces acquire a negative charge, enabling them to form aggregates with positively charged titanium dioxide. This interaction enhances the adsorption of titanium dioxide (TiO2) by the MPs. Concurrently, the presence of TiO2 is found to increase the surface roughness of the MPs, which in turn promotes greater adsorption of MPs by the quartz sand [6]. Consequently, following the environmental interaction between MPs and heavy metals, there is a subsequent alteration in both their physical and chemical properties. This change subsequently impacts the absorption of MPs by plants [7].

Compared with heavy metals, arsenic (As), as a metal, experiences more complex valence changes and is more susceptible to environmental factors. This complexity makes its research even more challenging [8]. Instead, the rapid expansion of industrial operations, including mining, smelting, leather tanning, chemical manufacturing, and the burning of fossil fuels, coupled with the widespread use of arsenic-rich pesticides and fertilizers, has led to the massive accumulation of arsenic in the water environment [9]. Thus, MPs and arsenic inevitably constitute a common pollutant scenario. In this context of co-contamination, the MPs of the polystyrene group are able to adsorb trivalent arsenic through hydrogen bonds, with an adsorption capacity that reaches 1 mg/g [10]. Arsenic enhances the negative charge on the surface of PS-MPs, facilitating the entry of more PS-MPs into carrot tissue [11], and the cell walls are compromised by the presence of heavy metals, enabling MPs with larger particle sizes to penetrate plant cells [11]. Polystyrene (PS) and polytetrafluoroethylene (PTFE) directly adsorb arsenic, which affects the arsenic adsorption sites on the root surface and inhibits the activity of the rice root system, thereby suppressing the absorption of arsenic by rice [12]. Additionally, these materials cause mechanical damage to the root system, leading to the generation of a significant amount of reactive oxygen species (ROS). This, in turn, inhibits the vitality and transpiration of the rice root system, reducing the plant’s capacity to absorb nutrients and water [13]. Consequently, this leads to a decrease in the photosynthetic capacity and biomass accumulation of rice. Previous research has indicated that the presence of MPs can reduce the absorption of arsenic in its trivalent form arsenite (As(III)) by rice seedlings and carrots cultivated through hydroponic methods. Current research on how As(V) influences the migration mechanisms of MPs within plant systems, as well as the long-term impacts of exposure to both As(V) and MPs on plant growth and metabolic pathways, remains inadequate.

Lettuce (*Lactuca sativa* L.), renowned as a significant vegetable crop with an extensive root system, is particularly vulnerable to the effects of environmental MPs [14]. Through hydroponic experiments with less interference factors, it is convenient to study the mechanism of the influence of arsenic on MPs in lettuce. This study also explores the toxic effects on lettuce’s physiological, biochemical, and metabolic processes under co-contamination. In light of this, the main focus of this study is to investigate (1) whether the addition of As(V) affects the migration and transformation of MPs in lettuce; (2) the impact of MP and As(V) co-contamination on physiological and biochemical indicators as well as the nutritional quality of lettuce; (3) the mechanism by which co-contamination affects plant metabolism and to elucidate the relevant molecular mechanisms. This study aims to establish the influence of common MPs on the migration and transformation of As(V) in the plant–water system and to reveal its main mechanisms, thereby filling the gaps in the biogeochemical cycle of As(V). This also helps to clarify the ecological and food safety risks of MP and As(V) co-contamination, and provides a theoretical basis for potential solutions to MP and As(V) pollution in complex agricultural environments.

## 2. Materials and Method

### 2.1. The Adsorption Effect of PS-MPs on As(V)

PS-MP spheres (5 μm) were provided by Qingdao ABET Instrument Technology Co., Ltd. (Qingdao, China). As(V) standard solution was supplied by Shanghai Anpu. Adsorption experiments were performed at 25 °C to investigate the adsorption of As(V) by PS over time. We mixed 50 mg of polystyrene (PS) with 50 mL of 50 mg/L As (V) solution in a 100 mL beaker and stirred the mixture at a speed of 1200 rpm. After 5, 15, 30, 60, 120, 240, 480, 720, 960, 1200, 1440, and 1920 min, we withdrew 0.5 mL of supernatant, removed impurities through a 0.45 μm water-soluble membrane, diluted it to an appropriate concentration, and determined the As (V) concentration of supernatant using an inductively coupled plasma mass spectrometer (ICP-MS, 7700X, Agilent Limited, Santa Clara, CA, USA).

Adsorption experiments were conducted at 15, 25, and 35 °C to study the effect of different temperature conditions on the adsorption of As(V) by polystyrene. We added 100 mL of As(V) solution with initial concentrations of 10, 20, 30, 40, and 50 mg/L to a beaker; then, we added 50 mg of polystyrene and agitated it at 400 rpm for 24 h. Impurities were removed from the solution using a 0.45 μm aqueous-phase filter membrane, and it was diluted to an appropriate concentration. We determined the As(V) content using an ICP-MS (7700X, Agilent Limited, USA). For Fourier transform infrared spectroscopy (FTIR) analysis, we took 20 mg of the prepared sample, used the potassium bromide (KBr) pellet method (Nicolet iS10, Thermo Fisher, Waltham, MA, USA), and analyzed it using Spectrum v5.0 software.

### 2.2. Culture of Lettuce and pH Determination of Nutrient Solution

Lettuce was selected as the subject of this study. the seeds were disinfected with 75% alcohol for 3 min, rinsed with ultra-pure water three times, and soaked in ultra-pure water for 30 min. The lettuce seeds were cultivated in vermiculite (at room temperature, in the dark) until they reached the 4–5 true leaf stage; then, we transferred the uniformly sized lettuce seedlings to Hoagland solution for 10 days of cultivation, with the cultivation solution preparation and conditions referring to previous research methods [15]. Hoagland solution powder was provided by Qingdao Haibo Biological Company (Qingdao, China). The Hoagland solution volume was set at 100 mL, and the solution was changed every 10 days to maintain the initial PS-MP and As(V) exposure concentration. The daily light cycle was 14 h at a photosynthetic photon flux density of 350 μmol/m^2^·s, with day/night temperatures of 25/20 °C and a relative humidity of 60%. Ultimately, we selected uniformly sized lettuce seedlings for the contamination exposure experiment. Before the lettuce was poisoned, the pH of the cultivation solution of each treatment group (for detailed information on the groups, please refer to the Supplementary Material) was adjusted to 6.0 with NaOH as the initial pH. Cultivation solution was collected from each treatment group 10, 20, and 30 days after lettuce contamination poisoning and measured with a pH meter.

To investigate the effects of different PS-MPs initial addition concentrations on the growth of lettuce, 5 mg·L^−1^ and 20 mg·L^−1^ were set as the microplastic stress conditions (denoted as P5 and P20, respectively, and P stands for PS-MPs), and plants not exposed to MPs were used as the blank control group (CK). To investigate the effect of arsenic on microplastic stress in lettuce, As(V) was added to Hoagland’s solution. This concentration refers to the concentration that has an effect on the plant [16]. A concentration below the point of exceedance and a concentration at the point of exceedance of 1 mg-L^−1^ and 10 mg-L^−1^ As(V) additions (expressed as P5V1, P5V10, P20V1, and P20V10, with V representing As(V)) were added to the plants, including CK, for a total of 9 treatments. Each treatment was repeated 3 times, for a total of 27 pots, with one lettuce per pot, and individually named, as shown in Table S1. The solution was renewed every 10 d and was selected for the effects on graduate vegetable tissues.

### 2.3. Exposure Response of Lettuce to PS-MPs

Transmission electron microscopy (TEM, Hitachi-7800, Tokyo, Japan) was utilized to observe changes in the ultrastructure of leaf cells after 30 days of exposure. Fresh lettuce roots were immersed in pre-cooled fixative (2.5% glutaraldehyde), and root tissues were cut into thin slices (0.5 × 0.5 × 4 mm) with a razor blade. The root tissues were pumped until they were completely in the fixative and left to stand at −4 °C for 24 h. The cellular ultrastructure was observed with TEM at 80 kV. Fresh lettuce roots were immersed in prepared formalin–aceto-alcohol fixative (formalin (38% formaldehyde), glacial acetic acid, and 70% alcohol), and lettuce leaves and roots were cut into thin slices (0.5 × 0.5 × 4 mm) with a razor blade, immersed into the fixative, and then pumped to vacuum. The tissues were left to be completely immersed in the fixative. The treated samples were removed, placed on coverslips, and photographed (200×) on a laser confocal microscope (Zeiss, LSM880, Jena, Germany) to observe the distribution of MPs in lettuce leaf and root cells.

To further determine whether the abnormal spheres in the plant root system were PS-MPs, a separate set of control experiments using fluorescently stained PS spheres (5 μm) were conducted in this study. Laser confocal analysis (LCA) was performed on lettuce tissue on Day 10 of the infected PS-MPs using laser confocal scanning microscopy (LCAM).

In order to determine the growth performance of graduate vegetables at different growth stages, three lettuce samples were randomly selected to measure the root length and the fresh weight of roots and leaves, which were randomly selected at the early growth stage (up to the 10th day of growth, Day 10), middle growth stage (up to the 20th day of growth, or the rapid growth stage, Day 20), and harvest stage (up to the 30th day of growth, when growth tends to be stabilized, Day 30). A portable chlorophyll meter (SPAD-502, Minolta Camera, Osaka, Japan) was used to measure the SPAD (soil and plant analyzer development) value of leaves, and the average value was determined three times for each treatment group. Lettuce samples were collected for the determination of plant root length and root weight, respectively. CAT content was determined at 405 nm using a UV-visible spectrophotometer (T90, Purkinje General, Beijing, China) to study the effect of different treatments on the antioxidant system of lettuce. Similarly, SOD and POD activities were determined at 550 nm and 420 nm, respectively. Amino acids in lettuce leaves were analyzed using an amino acid analyzer.

Lettuce leaves and roots were digested with 10 mL of HNO_3_ to investigate the effect of PS on nutrient uptake in lettuce. ICP-MS (7700X, Agilent Limited, USA) was used to analyze nutrient concentrations [17]. Vitamin C, fiber, protein, and nitrite content in leaves were determined by following the instructions of the kit.

### 2.4. Metabolomics Analysis

Leaf metabolites were collected on Day 30 and immediately frozen in liquid nitrogen. They were ground with a high-speed grinder (IKA, Aachen, Germany) at 12,000 rpm for 5 min and collected in sterile test tubes. Ultrasonic-assisted liquid–liquid extraction was used to extract leaf metabolites. Subsequently, the solvent was removed by nitrogen drying and lyophilization. Leaf metabolites were detected by gas chromatography–mass spectrometry (GC-MS; 6890 N/5973, Agilent, USA) after derivatization with methoxamine hydrochloride and n-methyl-n(trimethylsilyl)trifluoroacetamide. Metabolite identification was performed with reference to the National Institute of Standards and Technology (NIST 16) mass spectrometry library, and metabolites with matching scores >70% were selected. Metabolic pathway analysis was performed using MetaboAnalyst version 4.0 (http://www.metaboanalyst.ca accessed on 1 January 2024) and the Kyoto Encyclopedia of Genes and Genomes (KEGG) library. Details of the metabolomics analysis are provided in the Supplementary Material.

### 2.5. Statistical Analysis

SPSS 26 was used to calculate the error limits and mean values of the data, and Origin 2024 software was used to plot and fit adsorption kinetics, adsorption isotherms, etc. All the lettuce experiments were performed in 3 parallels, and the results are given as the mean and standard deviation. SPSS 26 software was used to statistically analyze the data. Data were analyzed by one-way analysis of variance using Tukey’s test, with *p* < 0.05 as a statistically significant difference. Lettuce leaf metabolites with matching scores greater than 70% were selected. Data were imported into SIMCA 14.1 (Umetrics, Umeaa, Sweden). Principal component analysis (PCA) was then performed to determine the relationship between the treatment and control groups. Variable importance (VIP) values in the projections were calculated using orthogonal projection to the latent structure (OPLS-DA) model. Metabolic pathway analysis of metabolites was completed using the KEGG database, followed by metabolic pathway and enrichment analysis using the Metabolic Analysis Tool.

## 3. Results and Discussion

### 3.1. Adsorption of As(V) by PS-MPs

**Figure 1:**
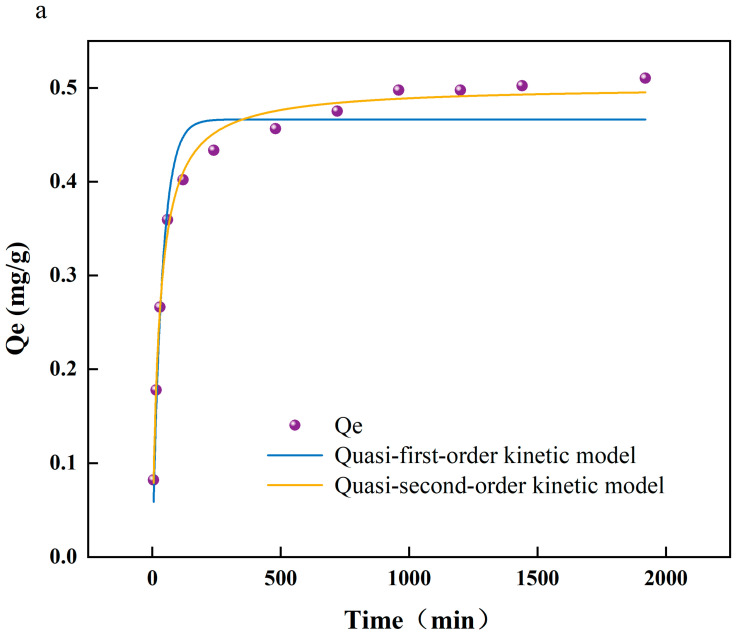

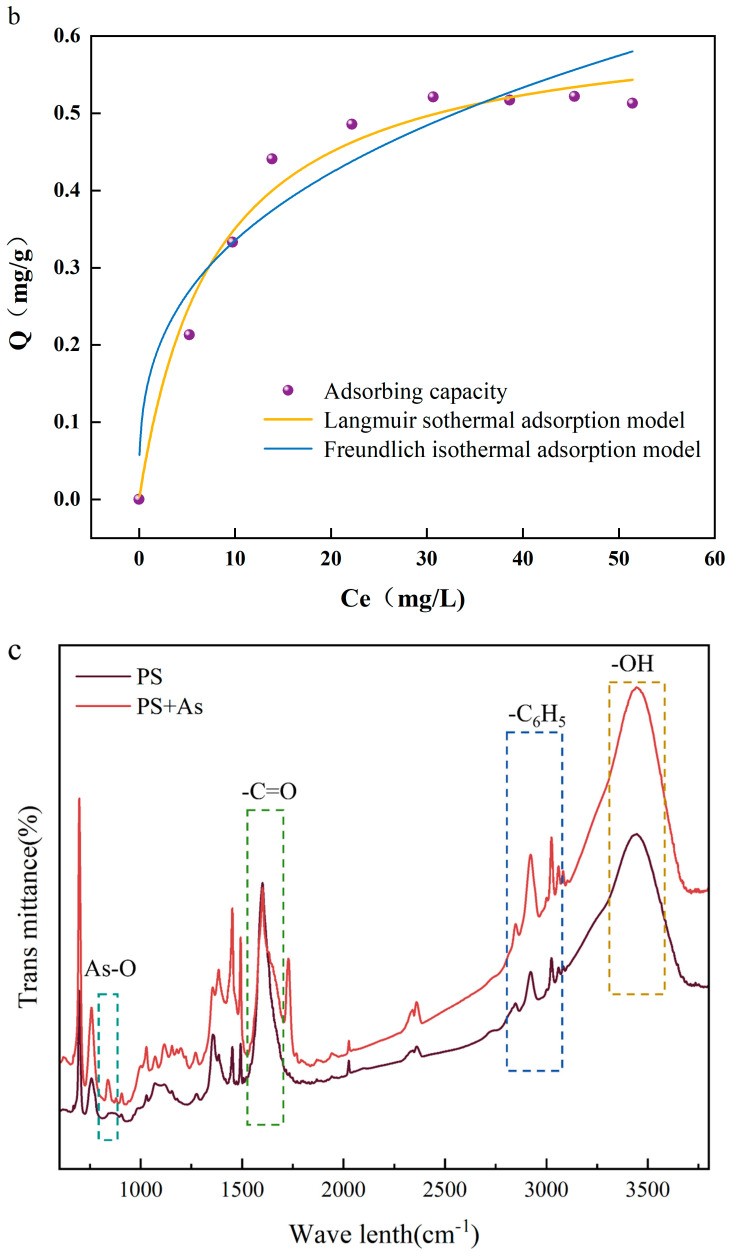
(**a**) Fitting curve of adsorption kinetics of arsenate (As(V)) adsorbed by polystyrene microplastics (PS-MPs); (**b**) isotherm of adsorption of As(V) by PS-MPs; (**c**) infrared spectroscopic analysis of adsorption of As(V) by PS-MPs and PS-MPs + As(V) at (288 K).

To achieve more profound comprehension of the interactions between arsenic and PS-MPs, as well as the underlying reaction mechanisms, this study employed a combination of adsorption kinetics, thermodynamic analyses, and Fourier transform infrared spectroscopy (FTIR) to scrutinize the chemical property alterations on the PS-MPs’ surfaces. Additionally, this research assesses the influence of environmental factors, including temperature and pH, on the adsorption efficiency. Figure 1a exhibits the kinetic modeling of As(V) adsorption on the surface of PS-MPs. A rapid increase in the adsorption of As(V) on PS-MPs (5 μm) was observed at the beginning of the experiment, and the growth rate was slowed down after 200 min until the adsorption equilibrium was reached at 800 min. The quasi-secondary kinetic model (R^2^ = 0.993) showed a better fit than the quasi-primary kinetic model (R^2^ = 0.954), which is the same as that of [18]. Therefore, it is concluded that the adsorption mechanism of As(V) on PS-MP surfaces is dominated by chemisorption. Figure S1 shows the adsorption of As (V) by PS-MPs at different temperatures (288, 298, and 308 K). It can be seen that the adsorption of As(V) by PS-MPs gradually decreases with the increase in temperature. A possible reason why the temperature inhibited the adsorption of As(V) by PS-MP particles was that when the temperature rose from 288 K to 308 K, the hydrogen bonds were broken, which led to a decrease in the adsorption efficiency of PS-MP particles to As(V) [19].

The relevant literature has shown that both the Langmuir and Freundlich models are suitable for describing the effect of temperature on adsorption capacity during adsorption, especially in heavy metal adsorption studies [20]. Figure 1b demonstrates the isothermal adsorption model for the adsorption of As(V) by PS-MPs. The maximum adsorption capacity (Q_max_) of PS-MPs was 0.521 mg/g, and the Langmuir model predicted a maximum adsorption capacity (Q_max_) of 0.627 mg/g, which was higher than the experimental value. This is because the Langmuir model assumes that the adsorption sites on the surface of PS-MPs are homogeneous, and this assumption is difficult to fully satisfy under experimental conditions. The isothermal adsorption process of As(V) adsorption by PS-MPs follows the Freundlich model to a large extent [21]. Discussions support that the adsorption of As(V) by PS-MPs is nonuniform surface multilayer adsorption. The results of thermodynamic analyses showed that changes in environmental conditions have an effect on the adsorption capacity, so the effect of temperature changes on the adsorption efficiency was investigated.

In order to understand the molecular level changes in the adsorption mechanism, the FTIR technique was applied to analyze the changes in the surface functional groups of PS-MPs. Figure 1c demonstrates the changes in surface functional groups before and after As(V) adsorption on PS-MPs. Before As(V) adsorption, the FTIR spectra of PS-MPs showed characteristic peaks corresponding to the O-H bond (3485.4 cm^−1^) and the C-H bond (2813.56–3101.21 cm^−1^). The characteristic peaks above 3000 cm^−1^ were generated by the phenyl vibrations on PS-MPs. PS-MPs originally have no oxygen-containing functional groups, and the oxygen-containing functional groups measured on their surfaces may originate from surface oxidation during ball milling [22]. When As(V) was adsorbed on the PS-MPs, the characteristic peak of As-O was observed near 838 cm^−1^, indicating the successful adsorption of arsenic on the PS-MPs’ surfaces [12]. The increased energy required for the vibration of the absorption peaks associated with the hydroxyl group vibration is a phenomenon related to the structure of As(V), which can interact with the -OH functional group on the surface of PS [23].

### 3.2. Absorption and Transport of PS-MPs by Lettuce

**Figure 2:**
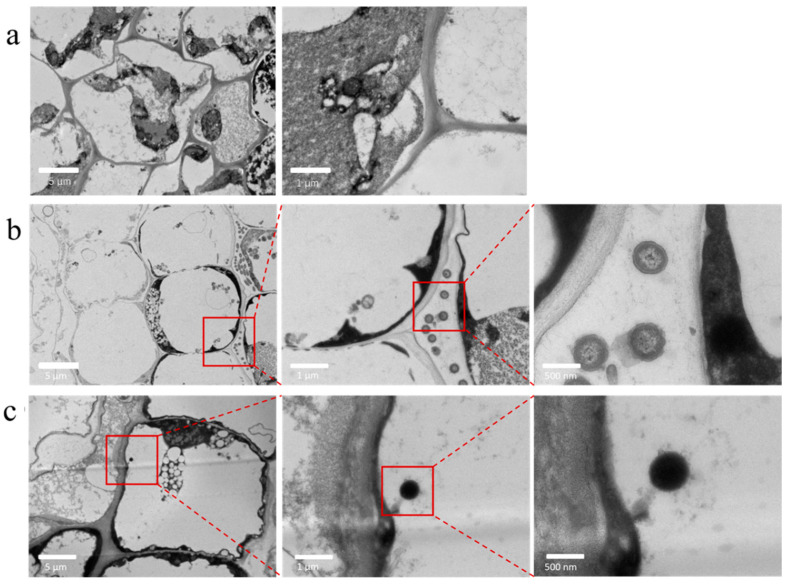
Transmission electron microscopy analysis of lettuce roots in different treatment groups ((**a**): PS-MPs; (**b**): PS-MPs + As(V); (**c**): PS-MPs + As(V)).

To determine whether lettuce grown in a hydroponic system could take up micrometer-sized MP spheres from the nutrient solution and translocate these particles from the roots to the leaves, and to identify uptake sites and pathways, Day 10 fresh lettuce root and leaf tissues were analyzed by TEM and confocal laser scanning microscopy (CLSM). As shown in Figure 2, the control lettuce root system of the CK group was analyzed by TEM, and no abnormal spherical particles were observed. In the P20 group, spherical particles appeared in the cell interstitial space, while in the P20V10 group, these abnormal spheres were found in the cytoplasm. Since the diameters of cell wall pores (3.5~5.0 nm) and intercellular connective filaments (midpoint 50~60 nm) were smaller than the diameter of PS-MPs beads (5 μm), it was hypothesized that the PS-MPs beads entered the meristematic tissues through the apical epidermis and entered the xylem through the extracellular body interstitials, confirming that the presence of arsenic may facilitate the penetration of MPs into the cell membrane. It was concluded that MPs are too large to pass through the physical barriers of intact plant tissues and therefore cannot be internalized directly into plant tissues. The Casparian zone of the root endodermis is thought to be a barrier to exocytotic movement that prevents chemicals and water from entering the root column. However, discontinuous regions are known to exist in the Casparian band of the root, through which unimpeded transport in the exocyst is possible. These zones are located where endodermal cells have not matured for root tip and secondary root formation [24]. Considering that these openings are a known pathway for plant pathogen or bacterial infection (sub-micron in width and up to a few micrometers in length), referred to as the fissure entry mode, these openings provide a pathway for the plant uptake of plastic particles [17].

In order to further determine whether the spheroids observed in the plant roots were PS-MPs, we performed a separate set of control experiments using fluorescently stained PS-MPs (5 μm). LCA was performed on lettuce tissue on Day 10 of the infected PS-MPs using LCAM, and the results were consistent with the TEM results. As shown in Figure 2a–c, there were no fluorescent spheres in the CK group, but PS-MP fluorescent spheres were found in the microplastic treatment group and were also found in the group with PS-MP and As(V) composite contamination, which illustrated that PS-MPs (5 μm) can enter the lettuce plant roots. Lettuce, as a leafy vegetable, was further analyzed by LCA of the leaves of the treatment group. It has been shown that when exposed to concentrations of 30 mg/L or more, microplastics can reach the central column of lettuce roots. On the cross-section of lettuce leaf veins under 40 mg/L treatment, the clustered structure of microplastics adhering to each other could be observed, and fluorescently labeled microplastics were observed [25].

Confocal images of lettuce cross-sections show that the PS luminescence signal is more confined to the vascular system of the root. Fluorescence was observed mainly along the cell wall and interstitial space, suggesting that 5 μm PS beads enter the cortical area through the epidermal intercellular space but are unable to penetrate the endothelium adjacent to the Casparis belt. A strong PS luminescence signal was detected in the fissures in the lateral root zone (50–100 mm from the tip), where the lateral roots pass through the endoderm and cortex, suggesting that these fissures are the primary entry point for submicron PS beads into the xylem of lettuce roots. The encapsulation of PS-MPs in the root crown mucus facilitates their penetration into the cell wall, allowing them to spread through the apical meristem, which is highly porous due to active cell division [26]. However, the diameter of the cell wall pores (3.5–5.0 nm) and intercellular interconnect filaments (midpoint of 50–60 nm) is smaller than the 5 μm diameter of the PS beads used in this study. This finding is consistent with Lou et al., that the physical location of the aplasophatic bypass is not only in the region where the lateral root emerges but also in the lateral root itself. Beads were observed to penetrate the xylem of lateral roots, as well as the primary roots of lettuce, and the transpiration pull of the plant plays an important role in the absorption and transport of plastic beads, suggesting that the increase in the transpiration rate enhances the uptake of beads by plants [27]. Usually, MPs accumulate on the surface of the root system through hydrophobic interaction and subsequently enter the plant under the transpiration pull, while root secretion with a large amount of positive charge is not favorable for the accumulation of positively charged MPs at the root tip, so it is not easy for positively charged MPs to enter the plant, but due to the surface of the cell membrane having more negatively charged regions, which are more prone to interact with positively charged MPs, positively charged MPs are more toxic. However, since the surface of the cell membrane has more negatively charged areas, it is easier to interact with positively charged MPs, so positively charged MPs are more toxic to plants [28,29].

### 3.3. The Rhizosphere Environment Was Affected by the Combined Pollution of PS-MPs and As(V)

**Figure 3:**
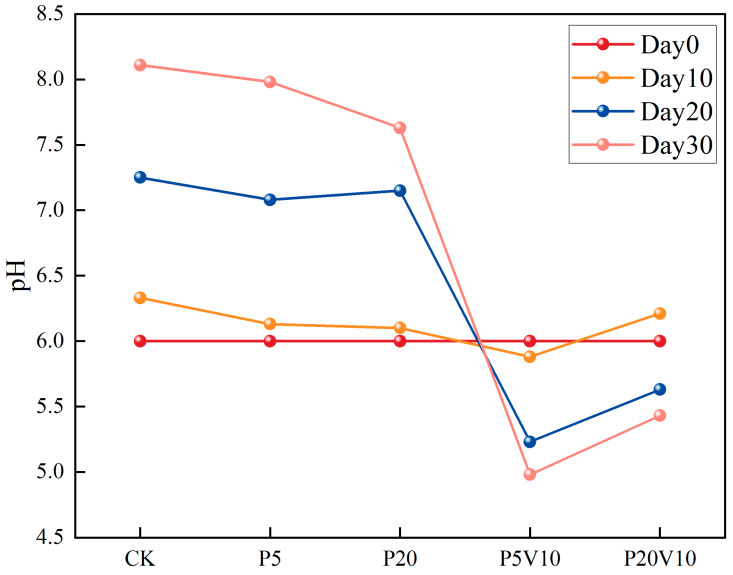
The change of pH on Day 10-Day 30 lettuce nutrient solution.

In order to investigate the effects of the composite pollution of PS-MPs and As(V) on the inter-root environment of lettuce, the specific effects of the composite pollutants on the growing environment of lettuce were assessed by determining the pH of lettuce culture solution in different treatment groups during a period of 10–30 d. It has been pointed out that when nitrate nitrogen fertilizer is applied, the nitrogen absorbed by the root system is dominated by nitrate nitrogen, and the inter-root of the plant releases OH^-^ and HCO^3−^, which leads to the elevation of inter-root pH [30]. As depicted in Figure 3, following the transplantation of lettuce into the culture solution, the pH in the PS-MP-exposed solution did not exhibit significant changes by Day 10 relative to the CK. Moreover, a decreasing trend with the increment of PS-MP concentration was observed, suggesting that low levels of PS-MPs had a minimal impact on plant root exudation. The PS-MPs influenced the pH, redox potential, nutrient content, conductivity, and organic matter of the rhizosphere environment [31]. Compared to the PS-MP treatment group, the acidification was further intensified by the introduction of low concentrations of As(V). By Day 20, the trend in pH reduction was more pronounced. As the lettuce grows, the pH in treatments P5V10 and P20V10 showed a downward trend, whereas the pH in the control group (CK) continued to increase significantly. This suggests that treatments P5V10 and P20V10 had a more substantial impact on pH, and this effect might have intensified over time. By Day 30, the pH of CK group escalated from 6.0 to 8.11. Upon the introduction of PS-MPs alone, the root systems sustained physical damage in the P20 group, leading to substantial secretions and a notable reduction in the nutrient solution’s pH to 7.63. The co-addition of As(V) further intensified the pH decrease, with the P20V10 group experiencing a drop of up to 33.0%.

To cope with adverse conditions, plants adapt to stressful environments by modulating metabolic secretions and their own life activity processes. Lettuce roots secrete organic acids, and this secretion significantly reduces the amount of cations that are exchanged and accumulated in the rhizosphere, which can alleviate the toxic effects of heavy metals on plants. Therefore, the secretion of organic acids has been recognized as one of the most effective external mechanisms for mitigating the toxins of heavy metals [32]. Plants have the capacity to modify the redox conditions, pH levels, and organic matter content within the rhizosphere. These alterations, in turn, influence the chemical speciation and mobility of trace elements, impacting their bioavailability and subsequent uptake by plant roots [33]. Plants also take up large amounts of insoluble nutrients from the inter-root and acidify the inter-root by excreting H^+^ in exchange for cations and emitting organic acids and CO_2_ [34]. These results are consistent with the decrease in inter-root pH that we observed.

### 3.4. Changes in Growth State and Oxidative Stress of Lettuce

**Figure 4:**
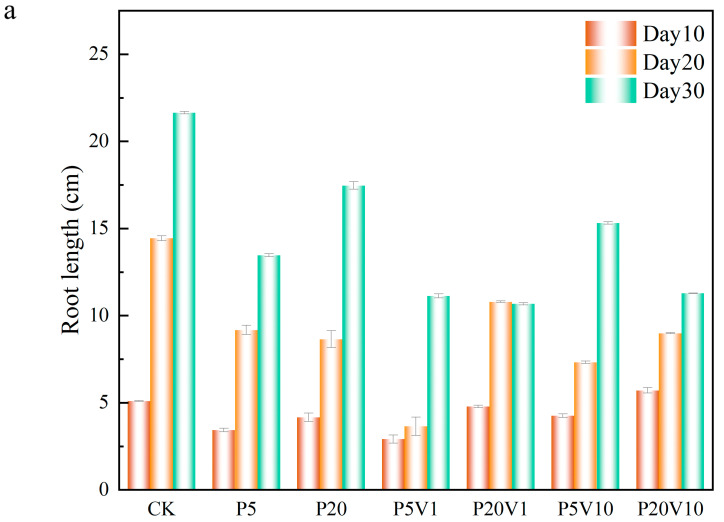

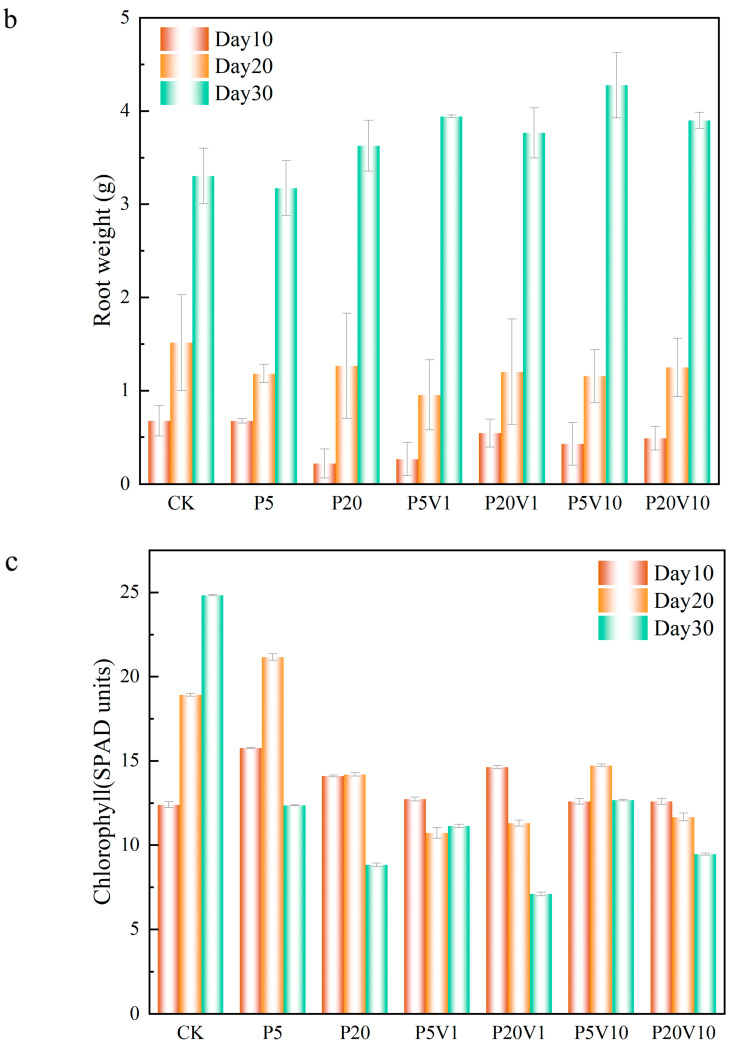

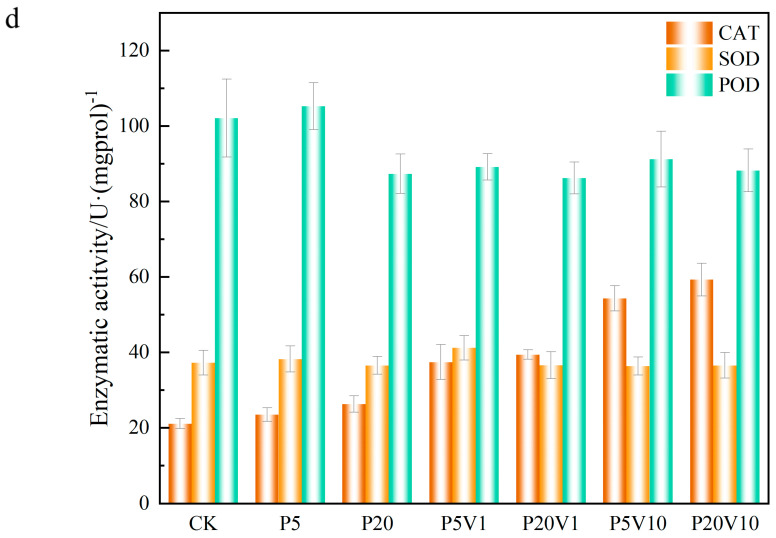
Growth performance of lettuce at different growth stages ((**a**): root length, (**b**): root weight, (**c**): chlorophyll); (**d**): changes in CAT, SOD, and POD of lettuce in different treatment groups on Day 30.

In order to investigate the role of PS-MP and As(V) composite pollutants on lettuce development, this study evaluated the detailed effects of these composite pollutants on lettuce growth by comparing lettuce root lengths, the fresh weight of roots and leaves, and leaf chlorophyll contents under different treatment conditions within different time periods (Day 10–Day 30). The root structure of a plant is a key factor in its productivity, and the healthy development of the root system is critical in shaping an ideal root system and may have profound effects on the growth and development of the plant throughout its life [35]. As shown in Figure 4a, on Day 10, compared to the CK group, in the PS-MPs treatment groups, root length increased 10.7% (0.93 cm, P20) and 32.9% (1.67 cm, P5), indicating that the addition of PS-MPs had a positive effect on lettuce root growth. Meanwhile, in the compound pollution treatment, on Day 10, the root length of each treatment group showed an overall decreasing trend over the period. On Day 20, the plant growth was similar to Day 10. On Day 30, the lettuce root length of P5 and P20 showed a significant decreasing trend, in which P20 and P20V1 were reduced by 64.4% (4.18 cm) and 71.4% (10.98 cm), respectively, in comparison, suggesting that As(V) would exacerbate the toxic effects of PS-MPs on plants, which is consistent with previous reports [36]. The root length of lettuce in the P20V10 treatment was lower than that of the CK group throughout the observation period, indicating that the combination of As(V) and PS-MP treatments had a more pronounced effect on lettuce growth.

On Day 10, compared to P5, the range of the root weight increase in the co-exposure treatment group expanded to 24.3% (P5V1)–34.8% (P5V20). This indicates that the addition of As(V) promotes the growth of roots. The lettuce root weight was 67.4% (0.455 g, day 10) and 6.4% (0.25 g, day 20) lower in the P20 group compared to CK. The root weight of lettuce compared to the single PS-MP-stressed group (P5 and P20) was elevated by 16.5% (1.1 g, P5V10) to 7.0% (0.272 g, P20V10). On Day 30, P5 root weight was close to that of CK, while the root weight of the P20 group increased by 9.8% compared with that of the CK, suggesting that PS-MPs inhibit root growth in the mid-growth period but do not have a significant negative effect in the long-term growth process. Compared to the P5 and P20 group, the root weight of the combined treatment group and the rest of the treatment groups increased compared to the CK group (3.3 g), which indicated that the addition of pentavalent arsenic promoted the growth of roots system compared with PS-MP stress alone. When the plant root system is damaged, stem cells are induced for repair, and more severe damage produces lateral roots, resulting in increased root weight [37].

The chlorophyll changes are shown in Figure 4c. On Day 10, the overall chlorophyll of lettuce in the group with PS-MP addition alone showed an increasing trend, and the chlorophyll concentration of P20 was lower than that of P5, which increased by 13.34% (1.3 SPAD units, P20) and 27.08% (3.37 SPAD units, P5), respectively, compared with CK, indicating that PS-MPs had a certain positive effect. This is consistent with previous studies that reported significant changes in chlorophyll a, chlorophyll b, and carotenoids in wheat leaves exposed to PS-MPs [36]. On Day 20, under the combined contamination of PS-MPs and As(V), the chlorophyll content of all treatment groups was lower than that of the CK group, and the inhibitory effect of high PS-MP concentrations on chlorophyll was more strong. The chlorophyll content of lettuce in the P20V1 and P20V10 groups decreased by 30.7% and 18.9%, respectively, indicating that the effect of PS-MPs was the main factor affecting chlorophyll in the composite treatment, and the presence of As(V) exacerbated this effect. For different PS-MP concentrations, these changes showed different trends, and the improvement of plant photosynthetic pigments by PS-MPs was significant when the microplastic concentration was low (0–50 mg/kg) [38]. Cell wall pores could adsorb MPs with a particle size of 5–50 nm, causing the blockage of vesicle pores; meanwhile, MPs would accumulate toward the surface of root hairs, inhibiting plant nutrient uptake from the growth medium and affecting plant growth and development. On Day 30, all treatments showed reductions in chlorophyll content relative to the control group, with the most significant reductions in the P20 and P20V1 treatments, which showed reductions relative to CK of 68.45% and 71.37%. This suggests that the effects of prolonged high concentrations of PS-MPs negatively affected the growth of lettuce, while the addition of As(V) exacerbated this effect. The chlorophyll content of the CK group was 24.83, which was an increase of about 30.51% compared to Day 20, indicating that the chlorophyll content of lettuce continued to grow. Micron-sized polyethylene plastic particles can significantly inhibit the growth and produce physiological and genotoxicity in fava beans, whereas nano-sized polyethylene MPs only inhibit the growth of fava beans at high concentrations (100 mg/L) [39].

In this study, the leaf oxidative system was measured in lettuce under PS stress on Day 30. As shown in Figure 4d, CAT content was elevated by 11.42%, 24.19%, 157.63%, and 180.57% in the P5, P20, P5V10, and P20V10 treatment groups, respectively, compared with CK. The addition of As(V) exacerbated the effect of PS-MPs on CAT, indicating that the CAT content in lettuce was determined by the content of added As(V). As shown in Figure 4d, the content of SOD in P5 and P20 changed less, and after the addition of As(V), P5V10 and P20V10 did not change much compared to P5 and P20, respectively, indicating that the effects of compound pollution and PS-MP stress alone on SOD were similar. SOD mainly removes superoxide ions (detoxifies O_2_^−^ to H_2_O_2_). Depending on the metal at the active site, SOD can be categorized into three types: copper-zinc superoxide dismutase (Cu/Zn-SOD), manganese superoxide dismutase (Mn-SOD), and iron superoxide dismutase (Fe-SOD) [40], whereas Mn-SOD is located in cellular mitochondria [41]. As(V) inhibited the accumulation of Mn in lettuce and decreased the activity of Mn-SOD in lettuce mitochondria. The inhibition of Mn-SOD activity could have cascading effects on the plant’s antioxidant system, leading to an accumulation of ROS, which can cause oxidative stress and damage to cellular components [42]. As shown in the Figure 4d, the POD content was greatly affected by the high PS-MPs content, with P20 reduced by 14.4% compared with CK. The POD content increased with the As(V) concentration under compound pollution, suggesting that PS-MPs caused different degrees of membrane damage, exacerbated the effect of PS-MPs on the active enzymes, and activated the antioxidant system of lettuce leaves. POD is widely present in plants and animals and can eliminate the toxicity of hydrogen peroxide, phenolics, and amines, whereas the dysregulation of both ionic homeostasis and antioxidant systems suggests that MP stresses push the lettuce beyond its tolerance range, thereby inhibiting normal growth. Many reports have investigated the response of these antioxidants to arsenic exposure [43,44]. Complex pollution stress disrupts plant cellular homeostasis and leads to hyperosmotic damage in lettuce, and osmoregulation is the main plant response to stress [45]. Proton pumps help plant cells maintain osmotic pressure by regulating the intra- and extracellular pH balance and ionic concentration, thus adapting to a wide range of environmental stresses [46]. The activation of the antioxidant defense system is also an important stress response, such as the accumulation of enzymatic antioxidants and osmolytes [47]. The dysregulation of both ionic homeostasis and antioxidant systems suggests that MPs stress lettuce out of its tolerance range, thus inhibiting normal growth [48]. Combined PS-MP and As(V) contamination increases CAT production and decreases SOD production for defense through the plant’s own redox system.

### 3.5. The Absorption of Nutrient Elements and the Change of Quality of Lettuce

**Figure 5:**
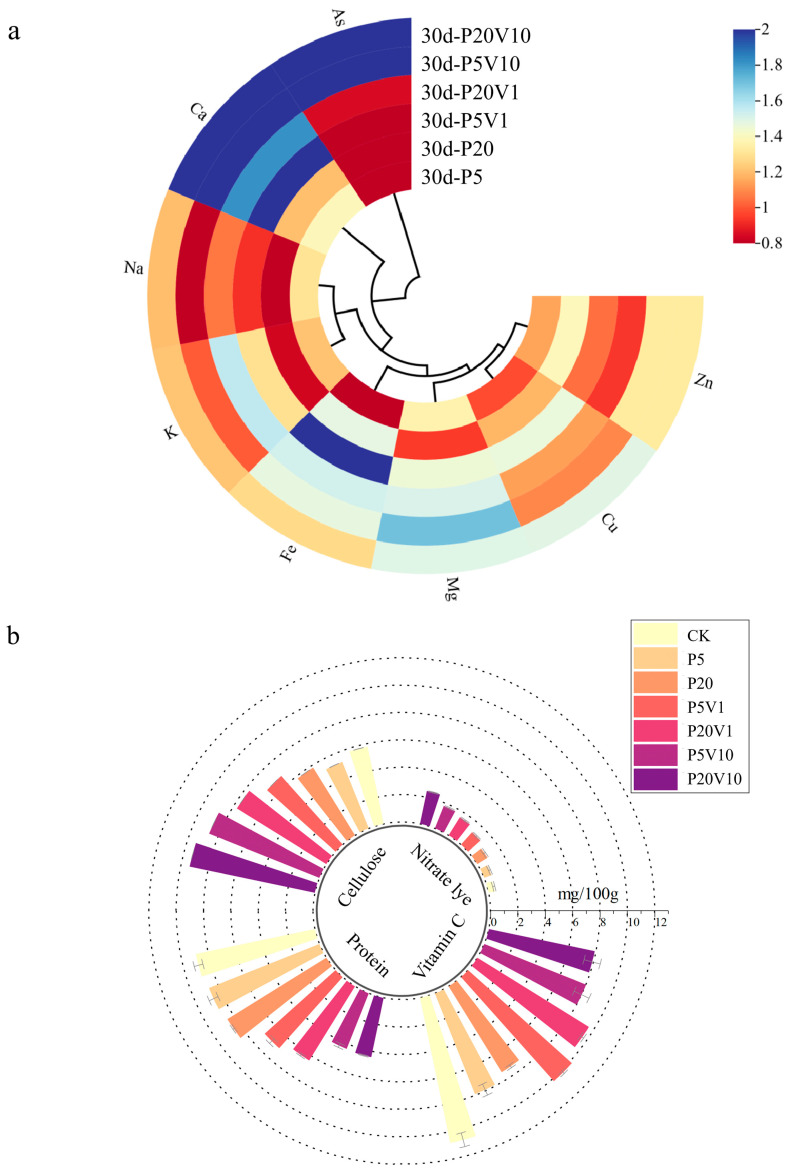

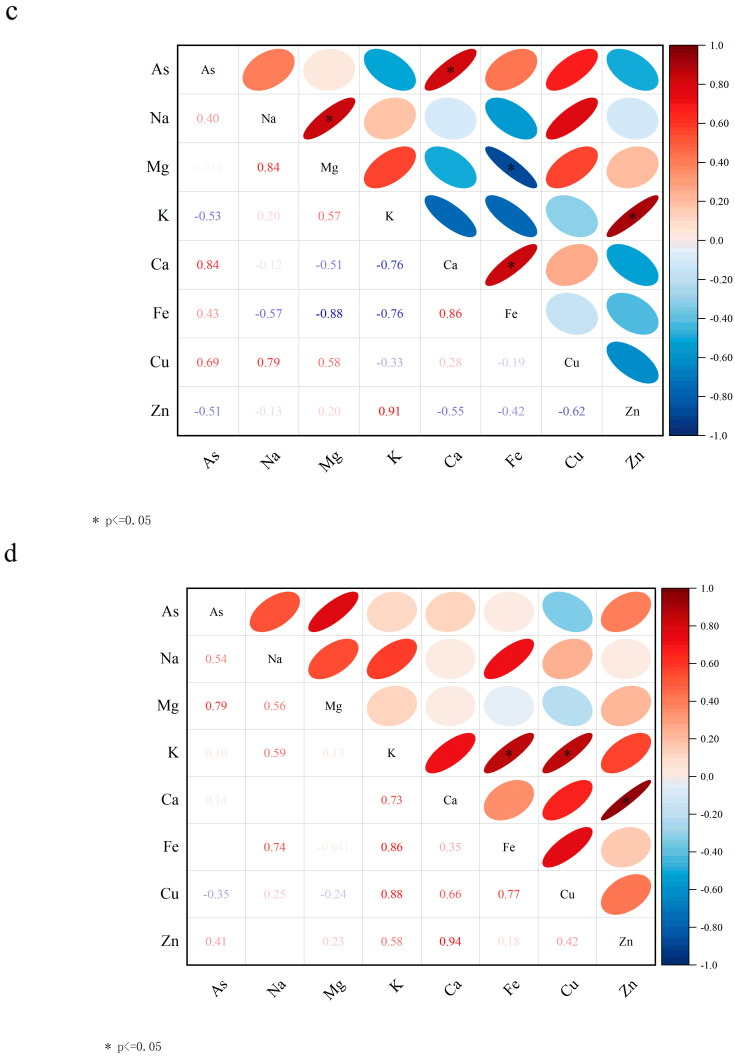
On Day 30, (**a**) changes in concentrations of plant nutrients between different treatment groups (relative blank group); (**b**) changes in nutrient index content of lettuce between different treatment groups; (**c**,**d**) correlation of nutrient element levels in leaves of P20 and P20V10.

The nutrient composition of Day 30 lettuce leaves and roots is shown in Figure 5a, where the addition of PS-MPs affected the uptake of nutrient elements and improved As(V) resistance in lettuce. Figure 5a shows the changes in leaf nutrient element (relative to the CK group) concentrations between different treatment groups on Day 30. Different concentrations of PS-MP treatments promoted calcium uptake by lettuce, the Ca content in plant leaves changed significantly, and the Ca content of lettuce in the composite treatment group decreased with increasing PS-MPs. PS-MPs increased the level of essential mineral calcium, which ultimately promoted plant growth. The content of Na under composite contamination increased with increasing PS-MPs concentration, reaching a maximum value of 1.20 in the P20V10 treatment group, which indicated that the addition of arsenic promoted the uptake of Ca Na and K by plants. Plants regulate cellular osmotic pressure through inorganic ion exchange (Na^+^ and K^+^) to maintain cellular swelling pressure and resist As(V) stress [49]. The Mg content of leaves did not change significantly under PS-MP stress alone, and the relative concentration of P20V1 reached the maximum value of 1.509. When only under PS-MP stress, the Cu content of lettuce leaves did not change much with the concentration of PS-MPs, which decreased by 23–30% compared with CK. The Cu content of leaves increased significantly under combined pollution, and the relative content of P5V10 reached 2.88. Composite contamination decreased the K content. K^+^ is an important element in regulating the osmotic pressure of plant cells, and through its concentration gradient inside and outside the cell, it helps to maintain the osmotic and expansion pressures of the cell, thus affecting the cell volume and growth status, indicating that MPs have a significant effect on the osmotic pressure of plant cells [50]. As(V) activates Ca^2+^ signaling in plant roots and interacts with the plasma membrane-localized As(V)/Pi transporter protein PHT1;1 via the calcium-dependent protein kinase CPK23, affecting As(V) uptake and plant tolerance to As(V) stress [51]. The most affected by stress are calcium and zinc. Ca plays a crucial role in cellular functions, for example, in reducing the softening and senescence of fruits [52]. Ca^2+^ is also involved in plant defense responses, regulating the expression of defense-related genes and hypersensitivity responses [53]. Zn is an important trace element involved in a variety of physiological functions in plants. Zn plays a structural and/or catalytic role in processes such as cell division, cell expansion, and protein synthesis [54].

Relative to P5 and P20, the cellulose content of lettuce leaves in the composite treatment increased with increasing concentrations of PS-MP and As(V) stresses, as shown in Figure 5b. The content of the CK group was 0.057 mg/g. P5 and P20 were reduced by 9.3% and 9.7%, respectively, compared with CK. Cellulose is the main component of the plant cell wall and plays a role in supporting cell morphology. However, high cellulose content will affect the taste of lettuce and reduce the quality of lettuce. Figure 5b shows that the cellulose content of lettuce leaves increased with increasing concentrations of PS-MPs and As(V). The protein content in lettuce leaves tended to decrease with increasing PS-MP and As(V) stress concentrations. Vitamin C is a reducing agent that enhances the activity of SOD enzymes, thus improving the body’s immunity and playing an important role in the body’s anti-cancer and anti-aging activities. The intake of vegetables with high nitrite content increases the risk of diseases such as gastrointestinal cancer [55]. As can be seen in Figure 5b, with the increase in stress concentration, there was an overall decreasing trend of vitamin C content and an overall increasing trend of nitrite content in lettuce leaves. It has been found that nitrite transporters play an important role in plant response to heavy metal stress, and plant uptake of NO_3_^−^ can improve tolerance to heavy metals. Thus, heavy metal stress can promote the uptake of nitrite in plants [56]. Combined contamination with PS-MPs and As(V) elevated cellulose and nitrite content and decreased protein and vitamin C content.

Figure 5c,d show the Pearson correlation analysis of nutrient levels between treatment group and CK lettuce leaves. As shown in Figure 5c, arsenic was negatively correlated with Na and K in the P20 treatment group. Plants regulate cellular osmotic pressure through inorganic ion exchange (Na^+^ and K^+^) to resist PS-MP stress. As shown in Figure 5d, As(V) was positively correlated with Ca, Fe, and Mn in the P20V10 group. Mn is a component of Mn-SOD that plays an important role in scavenging ROS [57]. This is consistent with the increase in SOD activity in leaves. Ca improves plant stress tolerance. Lettuce leaves treated with P20 and P20V10 were selected for mineral and nutrient analysis to compare the effects of As(V) addition to PS-MP addition alone. As(V) concentration was positively correlated with Ca and Cu concentrations in the leaves of the P20 group, and it was negatively correlated with Cu concentrations in the leaves of the P20V10 treatment group. Plants regulate cellular osmotic pressure through inorganic ion exchange (Na^+^ and K^+^) to maintain cell expansion pressure and resist arsenic stress. Arsenic is positively correlated with calcium, iron, and copper concentrations. Iron is a component of Fe-superoxide dismutase (Fe-SOD), which plays an important role in the scavenging of reactive oxygen species (ROS) [58]. It is consistent with the elevated SOD activity in leaves. Similar results were found in ferns, where arsenic reduced the essential mineral nutrients Mg and Ca, which ultimately inhibited plant growth [59]. Ca increased plant stress tolerance and was positively correlated with arsenic concentration in all three treatment groups. In summary, compound pollution caused emergency response mechanisms in plants through plant stress, which increased calcium uptake, and calcium ions were also involved in plant defense responses, regulating the expression of defense-related genes and hypersensitive responses [60].

### 3.6. Leaf Metabolomics Analysis

**Figure 6:**
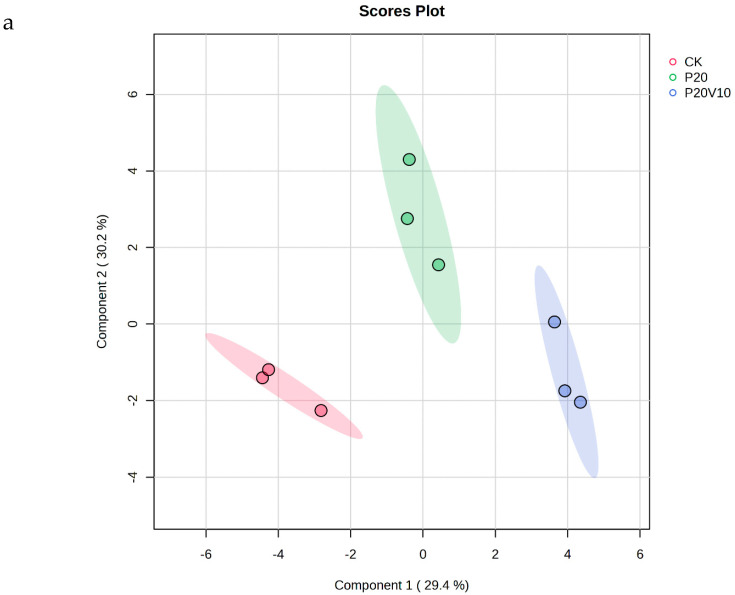

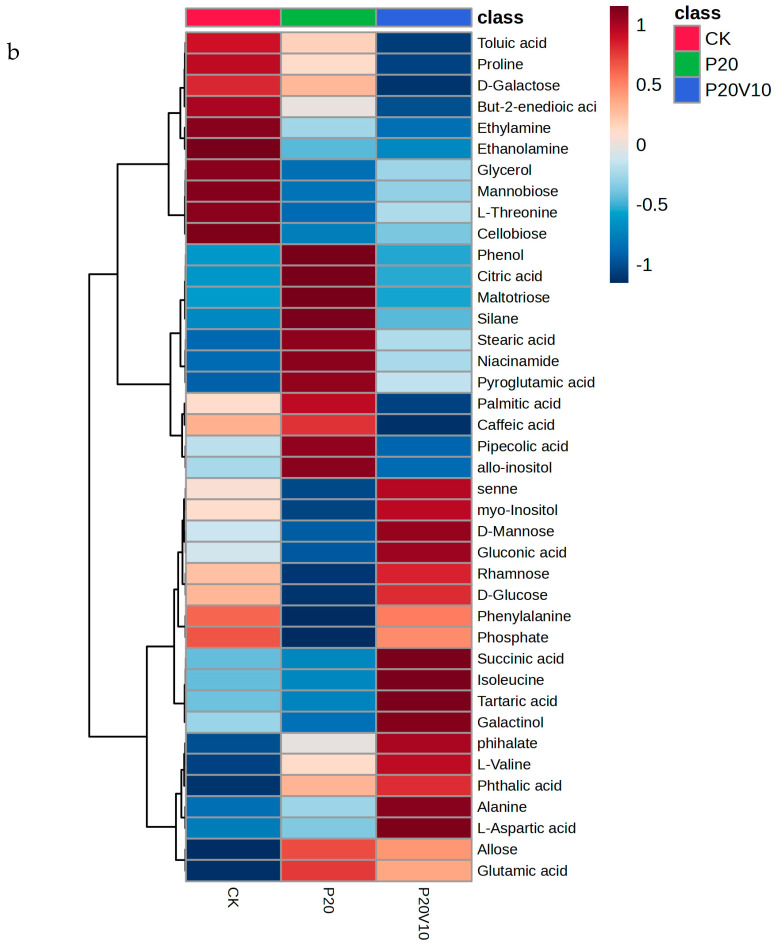

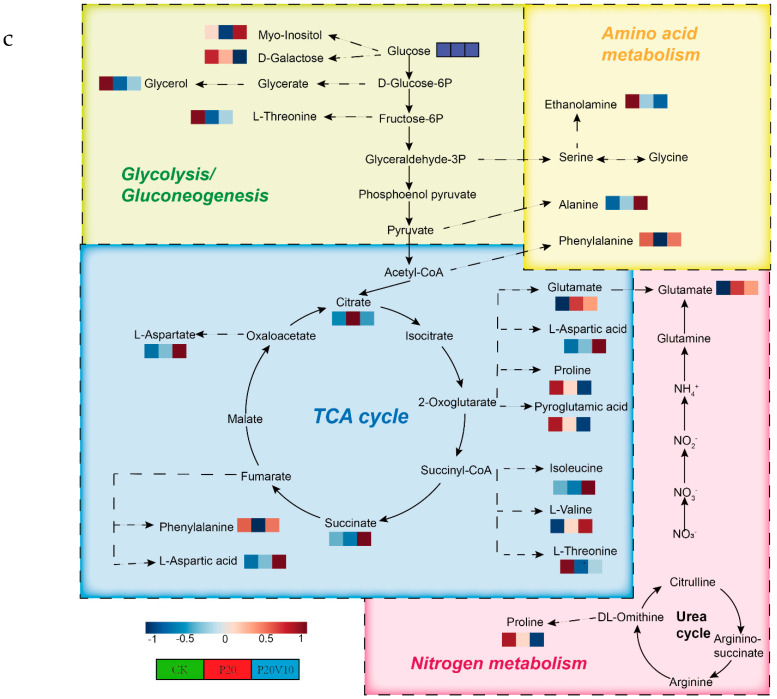
(**a**) PLS-DA scores of metabolites in lettuce leaves of CK, P20 and P20V10 treatment groups. (**b**) Heat maps of leaf metabolites in CK, P20, and P20V10 treatment groups. (**c**) Metabolic pathway map.

Plant metabolites are considered to be the end products of interactions between the genome and the environment and can express the mechanisms by which the environment affects the plant, and since metabolites are considered to be the end products of interactions between the genome and the environment [61], in this chapter, we determine the metabolites of Day 30 lettuce leaves to determine the molecular mechanisms of the toxicity of lettuce caused by combined contamination with PS-MPs and As(V). Due to the large number of treatment groups and the small variability among treatment groups with small concentration gradients, treatment groups with significant physiological and biochemical responses to PS-MPs (CK, P20, P20V10) and As(V) were selected for metabolite analysis to elucidate the molecular mechanisms of PS-MPs and As(V). To visualize the differences in lettuce metabolism across treatment groups, we performed PLS-DA based on the relative quantification of metabolites (Figure 6a). The results showed that P20 and P20V10 had significant effects on lettuce leaf metabolism compared with CK. As can be seen from the metabolic heatmaps in Figure S3, the changes in P20 metabolites mainly included galactose metabolism; nicotinic acid and nicotinamide metabolism; arginine biosynthesis; glutathione metabolism; the tricarboxylic acid (TCA) cycle; glycine, serine, and threonine metabolism; acetaldehyde and dicarboxylate metabolism; alanine, aspartate, and glutamate metabolism; phenylalanine metabolism; starch and sucrose metabolism; and lipid metabolism. The metabolic pathways were similar between the P20V10 and P20 treatments, mainly with down-regulation of arginine, proline, galactose, glycine, serine, and threonine metabolism. As can be seen in Figure 6b, the metabolites that were significantly up-regulated in P20V10 were involved in N and S metabolism compared to P20. In addition, the phenylalanine metabolism, starch and sucrose metabolism, and inositol phosphate metabolism metabolic pathways were up-regulated in P20V10. Significantly up-regulated metabolites in P20V10 were involved in the TCA cycle and sulfur metabolic pathways. Glycerophospholipids are constituents of cell or vesicle membranes, and their terminal ester groups are mainly composed of ethanolamine, choline, serine, or inositol [62]. The down-regulation of ethanolamine, an important signaling molecule, may be associated with the reduced catabolism of glycerophospholipids in plants, which helps to alleviate oxidative stress in plants in P20V10, which is consistent with the results in Figure 4d. The TCA cycle is essential for ATP production. The TCA cycle is important for ATP production, and As may affect ATP synthesis by influencing phosphorus (P), as As and P have similar chemical structures and As has an affinity for the transporter of P, which may affect ATP synthesis by competing with phosphate, leading to the inhibition of photosynthesis and reductions in biomass and chlorophyll [63]. TCA cycling, chlorophyll content, and leaf weight were significantly increased in P20V10 compared with P20. Myo-inositol-related metabolism was up-regulated in P20V10; myo-inositol and its derivatives are involved in the phosphatidylinositol signaling pathway, cell wall biosynthesis, growth hormone storage and transport, and the production of stress-related molecules [64]. The results are consistent with Figure 4c, which shows that the fiber content in P20V10 is lower than that in P20. Sulfur metabolism, the TCA cycle, and pyruvate metabolism are up-regulated in P20V10. The down-regulation of pyruvate metabolism led to a decrease in TCA-related carbohydrate metabolism in the plant, suggesting that the plant was weaker in maintaining the energy level under PS stress. PS and As stress showed synergistic regulation in lettuce, leading to significant up-regulation of the TCA cycle in P20V10.

Figure 6c illustrates the effects of PS-MPs and As(V) on lettuce leaf metabolism. PS-MPs had limited effects on differential metabolites [65]. In contrast, As(V) affected a major role in metabolite variation. As(V) significantly inhibited both the TCA cycle and the metabolism of glyoxylates and dicarboxylates, consistent with the disruption of the chlorophyll structure described above, resulting in the inhibition of photosynthesis and respiration and a decrease in biomass, plant height, and chlorophyll [60]. As(V) significantly inhibited alanine, aspartic acid, and glutamic acid metabolism. As(V) reduced the levels of most amino acids, such as proline and threonine. Higher proline production has been shown to be associated with increased plant metal tolerance [66]. Vitamin C levels were reduced compared to the control group. Phenolic compounds and vitamin C are metabolites of plant secondary metabolism and play an important role in defense against plant pathogens and abiotic stressors [67]. Phenolic compounds and ascorbic acid are metabolites derived from plant secondary metabolism and play an important role in defense against plant pathogens and abiotic stressors. Niacin content was also decreased. High concentrations of As(V) significantly inhibited the production of secondary metabolites in lettuce [68].

## 4. Conclusions

This study revealed the effects of combined PS-MP and As(V) contamination on lettuce growth and physiological responses. It was found that the combined effects of PS-MPs and As(V) had a significant effect on root length and biomass. In addition, co-contamination induced redox system responses in lettuce, including increases in CAT and decreases in SOD and POD. Lettuce enhanced its resistance to heavy metals by regulating histidine content in the body and chelating with As(V). Meanwhile, the down-regulation of inositol-related metabolism and reduced tyrosine synthesis affected carbohydrate metabolism in the plant, leading to a decrease in growth indices. The present study is important for understanding the potential effects of combined MPs and heavy metal pollution on crops. The findings suggest that PS-MPs not only act as carriers of As(V) and increase the uptake of As(V) by plants but may also further affect plant growth and development by influencing their metabolic pathways and signaling mechanisms. MPs are widely present in the environment and can be used as carriers of other pollutants and microorganisms to enter the ecological environment. High concentrations of microplastics may cause significant ecotoxicity to living organisms, affecting their survival. These findings are instructive for assessing the ecological risks of MPs in agroecosystems and developing corresponding risk management strategies. Although this study provides initial insights into understanding the effects of combined PS-MP and As(V) pollution on plants, there are still many questions that need to be further explored. Future research could focus on the following areas: first, in-depth study of the molecular mechanisms of PS-MP and As(V) interactions and how these interactions affect metabolic pathways and signaling in plants; second, to explore the effects of different types and sizes of MPs on plant growth and physiological responses, and whether these effects vary with MPs concentration; finally, to investigate the long-term adaptive responses of plants to combined MPs and heavy metal pollution and how these responses affect plant productivity and quality. These studies will contribute to a more comprehensive understanding of the environmental risks of MPs and heavy metals and provide a scientific basis for sustainable agricultural development.

## Data Availability

Dataset available on request from the authors.

## Supplementary Materials

The following supporting information can be downloaded at: https://www.mdpi.com/article/10.3390/toxics13020086/s1, Figure S1: Adsorption of PS-MPs on As(V) at different temperatures (288, 298 and 308 K); Figure S2: Laser confocal analysis of root (a. CK, b. PS-MPs, c. PS-MPs +As(V)) and of leaf (d. PS-MPs, e. PS-MPs +As(V)) in different treatment groups; Figure S3: Metabolic pathways of P20 (a,b), P20V10 (c,d) compared with CK. Up (a,c), down (b,d); Table S1: Experimental treatment group.

## References

[1] An Q., Zhou T., Wen C., Yan C. (2023). The effects of microplastics on heavy metals bioavailability in soils: A meta-analysis. J. Hazard. Mater..

[2] Gao Y., Gao W., Liu Y., Zou D., Li Y., Lin Y., Zhao J. (2024). A comprehensive review of microplastic aging: Laboratory simulations, physicochemical properties, adsorption mechanisms, and environmental impacts. Sci. Total Environ..

[3] Prajapati A., Narayan Vaidya A., Kumar A.R. (2022). Microplastic properties and their interaction with hydrophobic organic contaminants: A review. Environ. Sci. Pollut. Res..

[4] Zhang G.S., Liu Y.F. (2018). The distribution of microplastics in soil aggregate fractions in southwestern China. Sci. Total Environ..

[5] Li M., He L., Zhang M., Liu X., Tong M., Kim H. (2019). Cotransport and deposition of iron oxides with different-sized plastic particles in saturated quartz sand. Environ. Sci. Technol..

[6] Cai L., He L., Peng S., Li M., Tong M. (2019). Influence of titanium dioxide nanoparticles on the transport and deposition of microplastics in quartz sand. Environ. Pollut..

[7] Sandil S., Óvári M., Dobosy P., Vetési V., Endrédi A., Takács A., Füzy A., Záray G. (2021). Effect of arsenic-contaminated irrigation water on growth and elemental composition of tomato and cabbage cultivated in three different soils, and related health risk assessment. Environ. Res..

[8] Zhang S.X., Jiang X.Y., Tian Y., Peng X.Y., Liu Y.Y., Deng Z.W., Yin X.X., Wang L.H. (2022). Research Progress of Arsenic Bio-accessibility and Bioavailability in Soils and Foods. Asian J. Ecotoxicol..

[9] Cao Y., Zhao Y., Zhang X., Zhang W., Sun D., Feng H., Tang Y., Qiu R. (2022). Research progress on phytoextraction technology of arsenic contaminated soil in farmland. Chin. J. Environ. Eng..

[10] Dong Y., Gao M., Qiu W., Song Z. (2020). Adsorption of arsenite to polystyrene microplastics in the presence of humus. Environ. Sci. Process. Impacts.

[11] Dong Y., Gao M., Qiu W., Song Z. (2021). Uptake of microplastics by carrots in presence of As (III): Combined toxic effects. J. Hazard. Mater..

[12] Mamtimin X., Song W., Wang Y., Habibul N. (2023). Arsenic adsorption by carboxylate and amino modified polystyrene micro- and nanoplastics: Kinetics and mechanisms. Environ. Sci. Pollut. Res..

[13] Panda S.K., Gupta D., Patel M., Van Der Vyver C., Koyama H. (2024). Functionality of reactive oxygen species (ROS) in plants: Toxicity and control in Poaceae crops exposed to abiotic stress. Plants.

[14] Hua Z., Zhang T., Luo J., Bai H., Ma S., Qiang H., Guo X. (2024). Internalization, physiological responses and molecular mechanisms of lettuce to polystyrene microplastics of different sizes: Validation of simulated soilless culture. J. Hazard. Mater..

[15] Tang X., Wen J., Mu L., Gao Z., Weng J., Li X., Hu X. (2023). Regulation of arsenite toxicity in lettuce by pyrite and glutamic acid and the related mechanism. Sci. Total Environ..

[16] Wang Q., Wen J., Zheng J., Zhao J., Qiu C., Xiao D., Mu L., Liu X. (2021). Arsenate phytotoxicity regulation by humic acid and related metabolic mechanisms. Ecotoxicol. Environ. Saf..

[17] Li L.Z., Luo Y.M., Li R.J., Zhou Q., Peijnenburg W.J.G.M., Yin N., Yang J., Tu C., Zhang Y. (2020). Effective uptake of submicrometre plastics by crop plants via a crack-entry mode. Nat. Sustain..

[18] Li X., Cui Y., Du W., Cui W., Huo L., Liu H. (2024). Adsorption Kinetics and Mechanism of Pb(II) and Cd(II) Adsorption in Water through Oxidized Multiwalled Carbon Nanotubes. Appl. Sci..

[19] Qu Z., Sun F., Qie Z., Gao J., Zhao G. (2020). The change of hydrogen bonding network during adsorption of multi-water molecules in lignite: Quantitative analysis based on AIM and DFT. Mater. Chem. Phys..

[20] Khayyun T.S., Mseer A.H. (2019). Comparison of the experimental results with the Langmuir and Freundlich models for copper removal on limestone adsorbent. Appl. Water Sci..

[21] Nguyen T.K., Li X., Ren L., Huang Y., Zhou J.L. (2023). Polystyrene and low-density polyethylene pellets are less effective in arsenic adsorption than uncontaminated river sediment. Environ. Sci. Pollut. Res..

[22] Yang C., Guan J., Yang Y., Liu Y., Li Y., Fei Y. (2022). Interface behavior changes of weathered polystyrene with ciprofloxacin in seawater environment. Environ. Res..

[23] Tang S., Sun P., Ma S., Jin W., Zhao Y. (2023). The interfacial behaviors of different arsenic species on polyethylene mulching film microplastics: Roles of the plastic additives. J. Hazard. Mater..

[24] Wang Z., Zhang B., Chen Z., Wu M., Chao D., Wei Q., Xin Y., Li L., Ming Z., Xia J. (2022). Three OsMYB36 members redundantly regulate Casparian strip formation at the root endodermis. Plant Cell.

[25] Roy T., Dey T.K., Jamal M. (2023). Microplastic/nanoplastic toxicity in plants: An imminent concern. Environ. Monit. Assess..

[26] Shao Y.M., Yu X.X., Xu X.W., Li Y., Yuan W., Xu Y., Mao C., Zhang S., Xu J. (2020). The YDA-MKK4/M KK5-M PK3/MPK6 Cascade Functions Downstream of the RGF1-RGI Ligand-Receptor Pair in Regulating Mitotic Activity in Root Apical Meristem. Mol. Plant.

[27] Luo Y., Li L., Feng Y., Li R., Yang J., Peijnenburg W.J.G.M., Tu C. (2022). Quantitative tracing of uptake and transport of submicrometre plastics in crop plants using lanthanide chelates as a dual-functional tracer. Nat. Nanotechnol..

[28] Sun X., Yuan X., Jia Y., Feng L.-J., Zhu F.-P., Dong S.-S., Liu J., Kong X., Tian H., Duan J.-L. (2020). Differentially charged nanoplastics demonstrate distinct accumulation in Arabidopsis thaliana. Nat. Nanotechnol..

[29] Wang Y., Xiang L., Wang F., Wang Z., Bian Y., Gu C., Wen X., Kengara F.O., Schäffer A., Jiang X. (2022). Positively charged microplastics induce strong lettuce stress responses from physiological, transcriptomic, and metabolomic perspectives. Environ. Sci. Technol..

[30] Zhang H.Q., Zhao X.Q., Chen Y.L., Zhang L.Y., Shen R.F. (2019). Case of a stronger capability of maize seedlings to use ammonium being responsible for the higher 15N recovery efficiency of ammonium compared with nitrate. Plant Soil.

[31] Wang F., Zhang X., Zhang S., Zhang S., Sun Y. (2020). Interactions of microplastics and cadmium on plant growth and arbuscular mycorrhizal fungal communities in an agricultural soil. Chemosphere.

[32] Chen H.C., Wu K.J., Li R., Wang T., Zhou C., Ma W.C., Wei H. (2019). Effects of exogenous organic acids on the characteristics of Cd accumulation of Salix variegata under Cd stress. Acta Ecol. Sin..

[33] Ding N., Lin H., Zhang X.H., He Y., Yu G. (2022). Interaction mechanism between root secretion and rhizosphere microorganisms: A review. Chin. J. Soil Sci..

[34] Cai Y., Xu Y., Liu G., Li B., Guo T., Ouyang D., Li M., Liu S., Tan Y., Wu X. (2024). Polyethylene microplastic modulates lettuce root exudates and induces oxidative damage under prolonged hydroponic exposure. Sci. Total Environ..

[35] Sun C., Li D., Gao Z., Gao L., Shang L., Wang M., Qiao J., Ding S., Li C., Geisler M. (2022). OsRLR4 binds to the OsAUX1 promoter to negatively regulate primary root development in rice. J. Integr. Plant Biol..

[36] Wang Y., Xiang L., Wang F., Redmile-Gordon M., Bian Y., Wang Z., Gu C., Jiang X., Schäffer A., Xing B. (2023). Transcriptomic and metabolomic changes in lettuce triggered by microplastics-stress. Environ. Pollut..

[37] Matosevich R., Cohen I., Gil-Yarom N., Modrego A., Friedlander-Shani L., Verna C., Scarpella E., Efroni I. (2020). Local auxin biosynthesis is required for root regeneration after wounding. Nat. Plants.

[38] Gao M., Liu Y., Song Z. (2019). Effects of polyethylene microplastic on the phytotoxicity of di-n-butyl phthalate in lettuce (*Lactuca sativa* L. var. ramosa Hort). Chemosphere.

[39] Wang L., Liu Y., Kaur M., Yao Z., Chen T., Xu M. (2021). Phytotoxic Effects of Polyethylene Microplastics on the Growth of Food Crops Soybean (*Glycine max*) and Mung Bean (*Vigna radiata*). Int. J. Environ. Res. Public Health.

[40] Zhao H., Zhang R., Yan X., Fan K. (2021). Superoxide dismutase nanozymes: An emerging star for anti-oxidation. J. Mater. Chem. B.

[41] Qin S., He Y., Kuang J., Fan S., Huang P., Wang W. (2019). Research progress on the extraction and application of Mn-SOD. Sci. Technol. Food Ind..

[42] Mishra N., Jiang C., Chen L., Paul A., Chatterjee A., Shen G. (2023). Achieving abiotic stress tolerance in plants through antioxidative defense mechanisms. Front. Plant Sci..

[43] Zhang S., Bao Q., Huang Y., Han N. (2023). Exogenous plant hormones alleviate As stress by regulating antioxidant defense system in *Oryza sativa* L.. Environ. Sci. Pollut. Res..

[44] Peralta J.M., Travaglia C., Romero-Puertas M.C., Molina-Moya E., Furlan A., Castro S., Bianucci E. (2022). Decoding the antioxidant mechanisms underlying arsenic stress in roots of inoculated peanut plants. Plant Growth Regul..

[45] Wang B., Zhang H., Huai J., Peng F., Wu J., Lin R., Fang X. (2022). Condensation of SEUSS promotes hyperosmotic stress tolerance in Arabidopsis. Nat. Chem. Biol..

[46] Li Y., Zeng H., Xu F., Yan F., Xu W. (2022). H+-ATPases in plant growth and stress responses. Annu. Rev. Plant Biol..

[47] Ahanger M.A., Qin C., Begum N., Qi M., Dong X.X., El-Esawi M., El-Sheikh M.A., Alatar A.A., Zhang L. (2019). Nitrogen availability prevents oxidative effects of salinity on wheat growth and photosynthesis by up-regulating the antioxidants and osmolytes metabolism, and secondary metabolite accumulation. BMC Plant Biol..

[48] Wang C., Liu Y., Song Z., Gao M. (2021). Effects of microplastics and DBP on photosynthesis and nutritional quality of lettuce. J. Agro-Environ. Sci..

[49] Li J., Shen L., Han X., He G., Fan W., Li Y., Yang S., Zhang Z., Yang Y., Jin W. (2023). Phosphatidic acid–regulated SOS2 controls sodium and potassium homeostasis in Arabidopsis under salt stress. EMBO J..

[50] Lu Y., Yu M., Jia Y., Yang F., Zhang Y., Xu X., Li X., Lei J., Wang Y., Yang G. (2022). Structural basis for the activity regulation of a potassium channel AKT1 from Arabidopsis. Nat. Commun..

[51] Liu Y., Zhang Y., Wang Z., Guo S., Fang Y., Zhang Z., Gao H., Ren H., Wang C. (2023). Plasma membrane-associated calcium signaling regulates arsenate tolerance in Arabidopsis. Plant Physiol..

[52] Wang M., Wu Y., Zhan W., Wang H., Chen M., Li T., Bai T., Jiao J., Song C., Song S. (2024). The apple transcription factor MdZF-HD11 regulates fruit softening by promoting Mdβ-GAL18 expression. J. Exp. Bot..

[53] Tian W., Wang C., Gao Q., Li L., Luan S. (2020). Calcium spikes, waves and oscillations in plant development and biotic interactions. Nat. Plants.

[54] Ma T., Zhao L., Zhang J., Tang R., Wang X., Liu N., Zhang Q., Wang F., Li M., Shan Q. (2022). A pair of transporters controls mitochondrial Zn2+ levels to maintain mitochondrial homeostasis. Protein Cell.

[55] Ghasemi M., Ghasemi A., Alizadeh-navaei R. (2024). A Systematic Review and Dose—Response Meta-Analysis of the Association between Nitrate & Nitrite Intake and Gastroesophageal Cancer Risk. Nitric Oxide.

[56] Aluko O.O., Kant S., Adedire O.M., Li C., Yuan G., Liu H., Wang Q. (2023). Unlocking the potentials of nitrate transporters at improving plant nitrogen use efficiency. Front. Plant Sci..

[57] Kim J., Kim H., Yim B., Rhee J.-S., Won E.-J., Lee Y.-M. (2018). Identification and molecular characterization of two Cu/Zn-SODs and Mn-SOD in the marine ciliate Euplotes crassus: Modulation of enzyme activity and transcripts in response to copper and cadmium. Aquat. Toxicol..

[58] Gao W., He J., Chen L., Meng X., Ma Y., Cheng L., Tu K., Gao X., Liu C., Zhang M. (2023). Deciphering the catalytic mechanism of superoxide dismutase activity of carbon dot nanozyme. Nat. Commun..

[59] Zemanová V., Popov M., Pavlíková D., Kotrba P., Hnilička F., Česká J., Pavlík M. (2020). Effect of arsenic stress on 5-methylcytosine, photosynthetic parameters and nutrient content in arsenic hyperaccumulator *Pteris cretica* (L.) var. Albo-lineata. BMC Plant Biol..

[60] Chen J., Li L., Kim J.H., Neuhäuser B., Wang M., Thelen M., Hilleary R., Chi Y., Wei L., Venkataramani K. (2023). Small proteins modulate ion-channel-like ACD6 to regulate immunity in Arabidopsis thaliana. Mol. Cell.

[61] Zhang F., Chen W. (2021). Research progress of metabolomics in plant stress biology. Biotechnol. Bull..

[62] Liu J., Yang F., Mao S., Li S., Lin H., Yan X., Lin J. (2021). Advances in the physiological functions of plant lipids in response to stresses. Chin. J. Biotechnol..

[63] Shi W., Xu Y., Wu W., Zeng X.-C. (2022). Biological effect of phosphate on the dissimilatory arsenate-respiring bacteria-catalyzed reductive mobilization of arsenic from contaminated soils. Environ. Pollut..

[64] Song L., Wang Y., Guo Z., Lam S.M., Shui G., Cheng Y. (2021). NCP2/RHD4/SAC7, SAC6 and SAC8 phosphoinositide phosphatases are required for PtdIns4P and PtdIns (4, 5) P2 homeostasis and Arabidopsis development. New Phytol..

[65] Li M., Wei J., Wei B., Chen Z.-Q., Liu H.-L., Zhang W.-Y., Li X.-Y., Zhou D.-M. (2024). Metabolic response of lettuce (*Lactuca sativa* L.) to polystyrene nanoplastics and microplastics after foliar exposure. Environ. Sci. Nano.

[66] Li J., Song Y., Luan X., Gou Y., Xie T., Hong Y., Liu N., Su Y., Fu X., Zhong T. (2024). Endoplasmic reticulum adenylate transporter activity affects amino acid metabolism under photorespiratory conditions. Crop J..

[67] Paciolla C., Fortunato S., Dipierro N., Paradiso A., De Leonardis S., Mastropasqua L., de Pinto M.C. (2019). Vitamin C in plants: From functions to biofortification. Antioxidants.

[68] Rabelo M.C., Bang W.Y., Nair V., Alves R.E., Jacobo-Velázquez D.A., Sreedharan S., de Miranda M.R.A., Cisneros-Zevallos L. (2020). UVC light modulates vitamin C and phenolic biosynthesis in acerola fruit: Role of increased mitochondria activity and ROS production. Sci. Rep..

