# Supplementary material for: The Combined Toxic Effects of Polystyrene Microplastics and Arsenate on Lettuce Under Hydroponic Conditions
Source: Toxics. 2025 Jan 24;13(2):86. doi: 10.3390/toxics13020086 (PMC11860235; doi:10.3390/toxics13020086)
Supplement: Supplementary file 1 [file toxics-13-00086-s001.zip › toxics-3429829-supplementary.pdf]

# Supplementary Material

## The Combined Toxic Effects of Polystyrene Microplastics and Arsenate on Lettuce under Hydroponic Conditions

Li Mu[1,*,†], Ziwei Gao[1,†], Mengyuan Wang[1,†], Xin Tang[1,†], Xiangang Hu[2]

[1]Tianjin Key Laboratory of Agro-environment and Safe-product, Key Laboratory for Environmental Factors Control of Agro-product Quality Safety (Ministry of Agriculture and Rural Affairs), Institute of Agro-environmental Protection, Ministry of Agriculture and Rural Affairs, Tianjin 300191, China

[2]Key Laboratory of Pollution Processes and Environmental Criteria (Ministry of Education), Tianjin Key Laboratory of Environmental Remediation and Pollution Control, College of Environmental Science and Engineering, Nankai University, Tianjin 300350, China

Corresponding author: Li Mu;

E-mail: muli@caas.cn

Tel: +86-022-2361-1150

[†] These authors contributed equally to this work.

Table S1 Experimental treatment group

| | | |
|---|---|---|
| Without Poison | CK | |
| PS-MPs Alone | PS-MPs concentration 5 $mg \cdot L^{-1}$ | PS-MPs concentration 20 $mg \cdot L^{-1}$ |
| | P5 | P20 |
| Arsenates and PS-MPs Together | PS-MPs concentration 5 $mg \cdot L^{-1}$ | PS-MPs concentration 20 $mg \cdot L^{-1}$ |
| Arsenates concentration 1 $mg \cdot L^{-1}$ | P5V1 | P20V1 |
| Arsenates concentration 10 $mg \cdot L^{-1}$ | P5V10 | P20V10 |

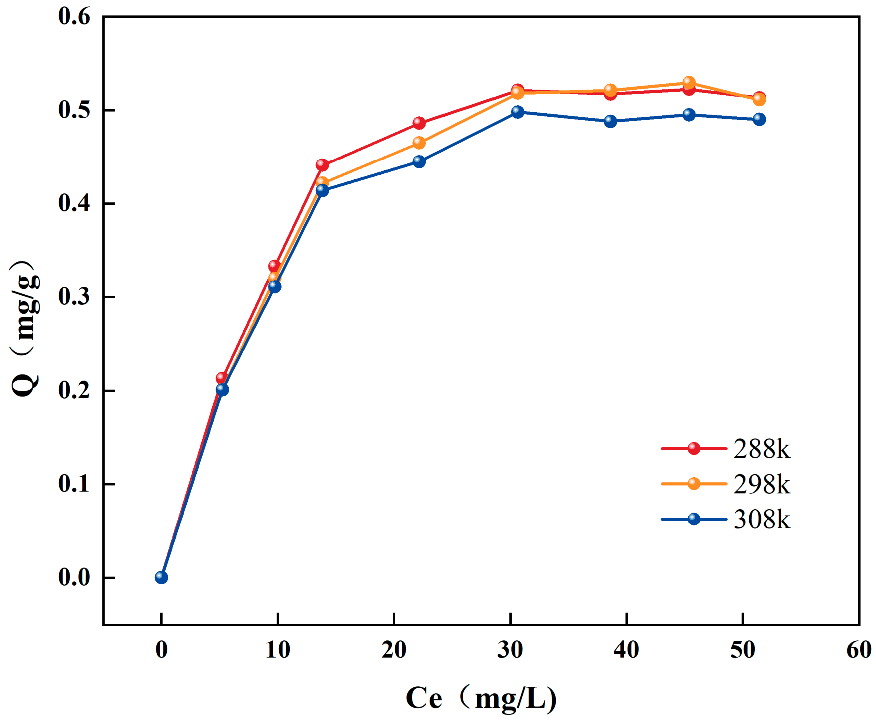


Figure. S1 Adsorption of PS-MPs on As(V) at different temperatures (288, 298 and 308 K)

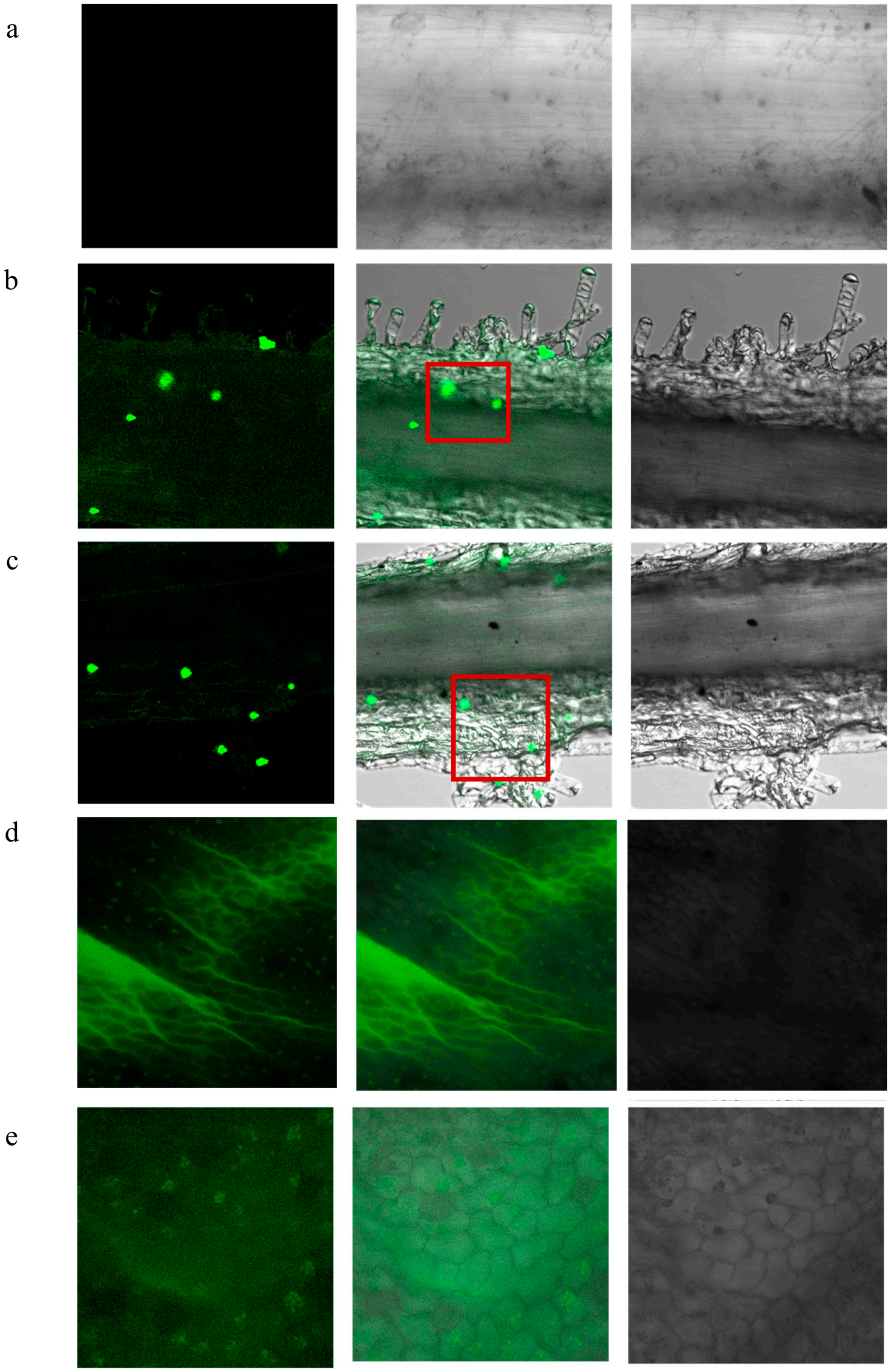


Figure. S2 Laser confocal analysis of root (a: CK, b: PS-MPs, c: PS-MPs +As(V)) and leaf (d: PS-MPs, e: PS-MPs +As(V)) in different treatment groups

a

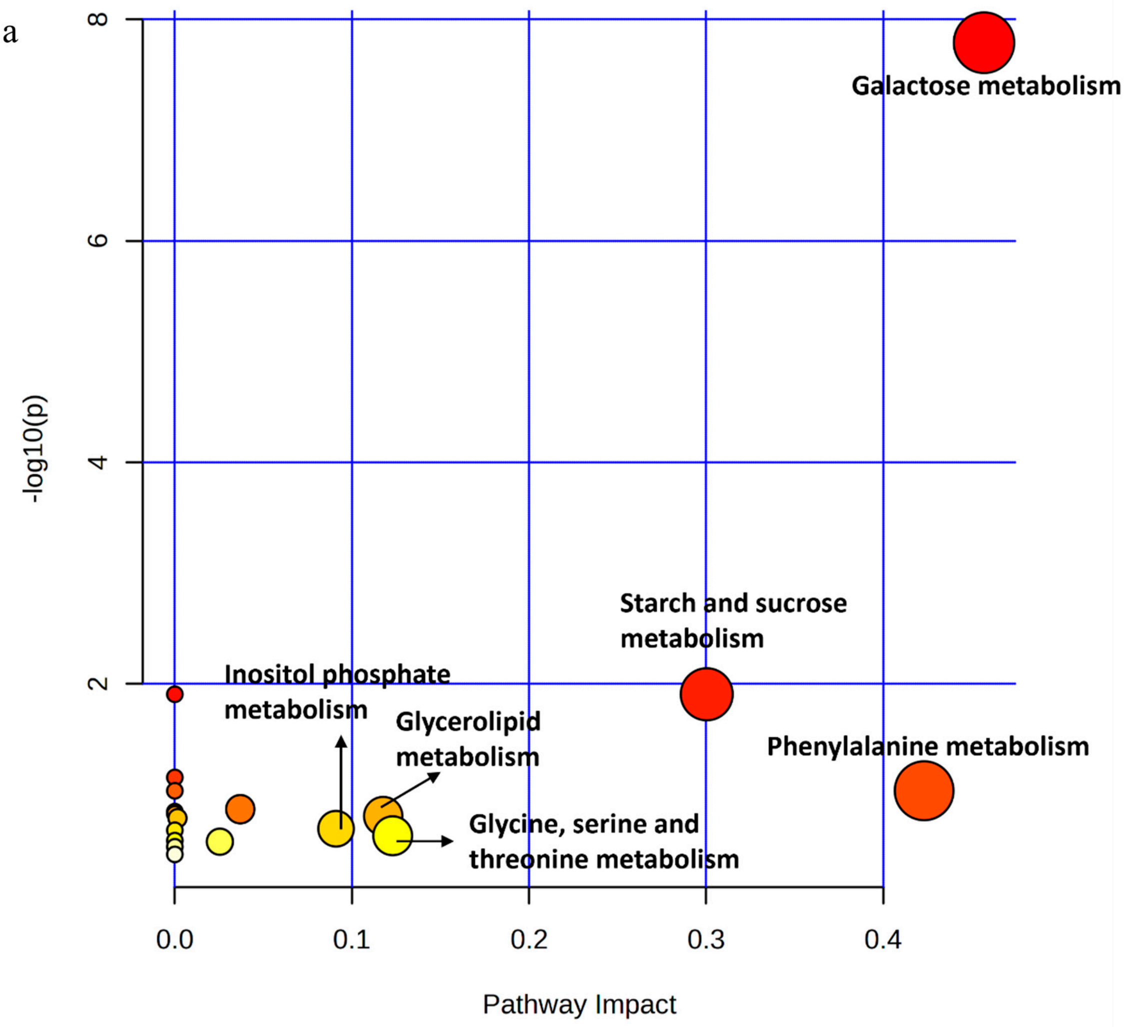

Galactose metabolism
Starch and sucrose metabolism
Inositol phosphate metabolism
Glycerolipid metabolism
Phenylalanine metabolism
Glycine, serine and threonine metabolism
-log10(p)
Pathway Impact

b

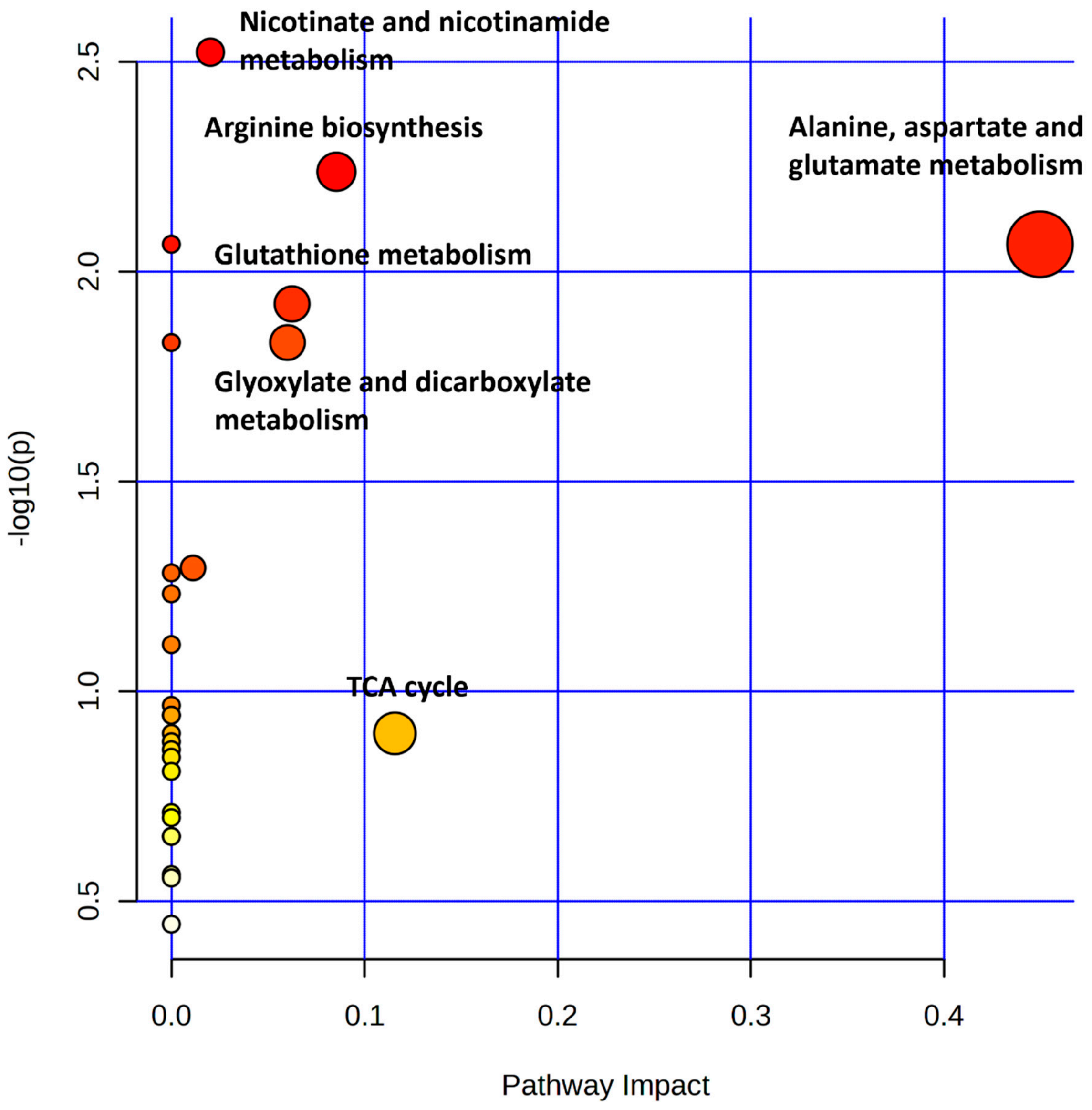
Nicotinate and nicotinamide metabolism
Arginine biosynthesis
Alanine, aspartate and glutamate metabolism
Glutathione metabolism
Glyoxylate and dicarboxylate metabolism
TCA cycle
-log10(p)
2.5
2.0
1.5
1.0
0.5
0.0
0.1
0.2
0.3
0.4
Pathway Impact

c

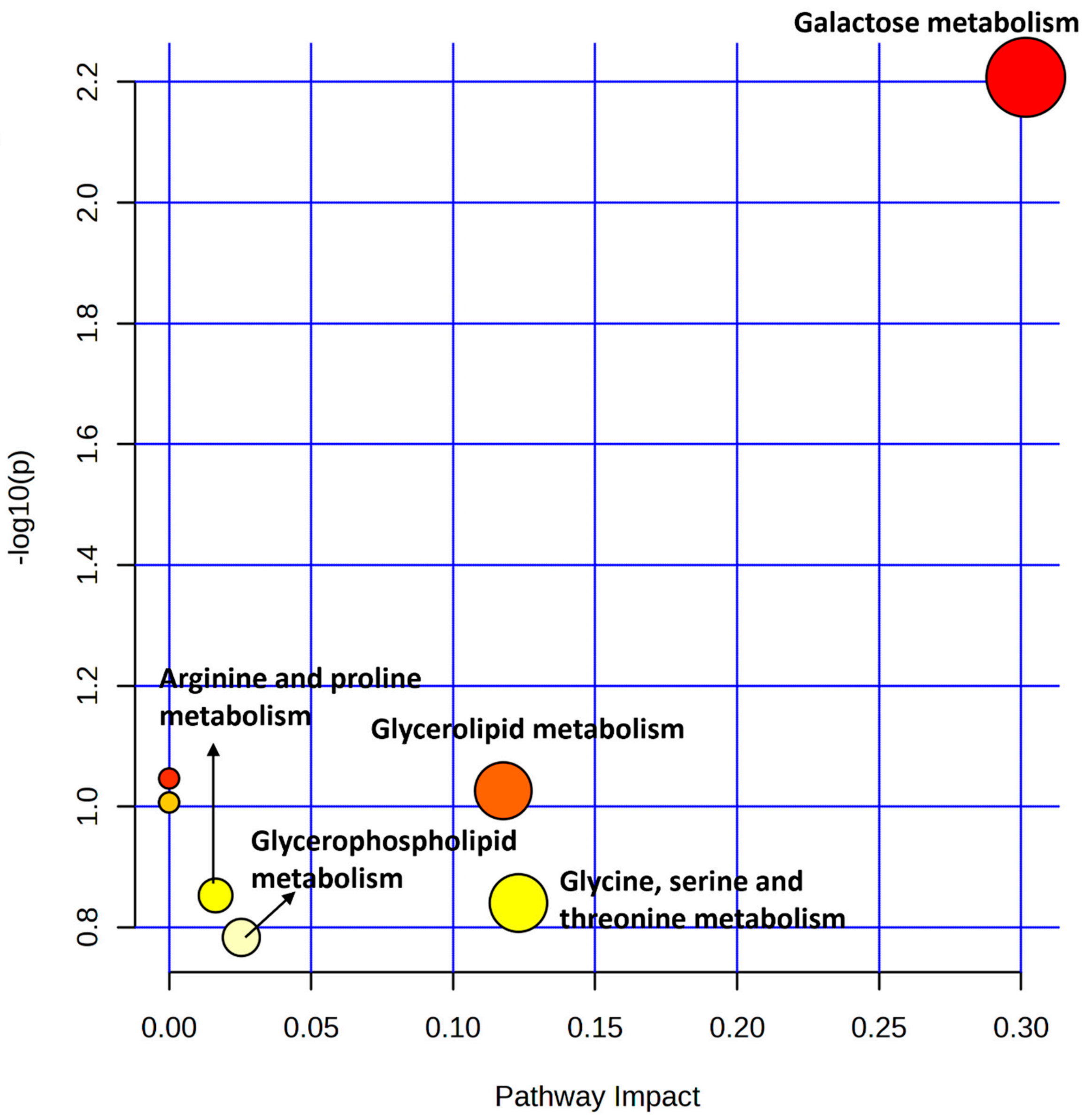

Galactose metabolism
Arginine and proline metabolism
Glycerolipid metabolism
Glycerophospholipid metabolism
Glycine, serine and threonine metabolism
-log10(p)
2.2
2.0
1.8
1.6
1.4
1.2
1.0
0.8
0.00
0.05
0.10
0.15
0.20
0.25
0.30
Pathway Impact

d

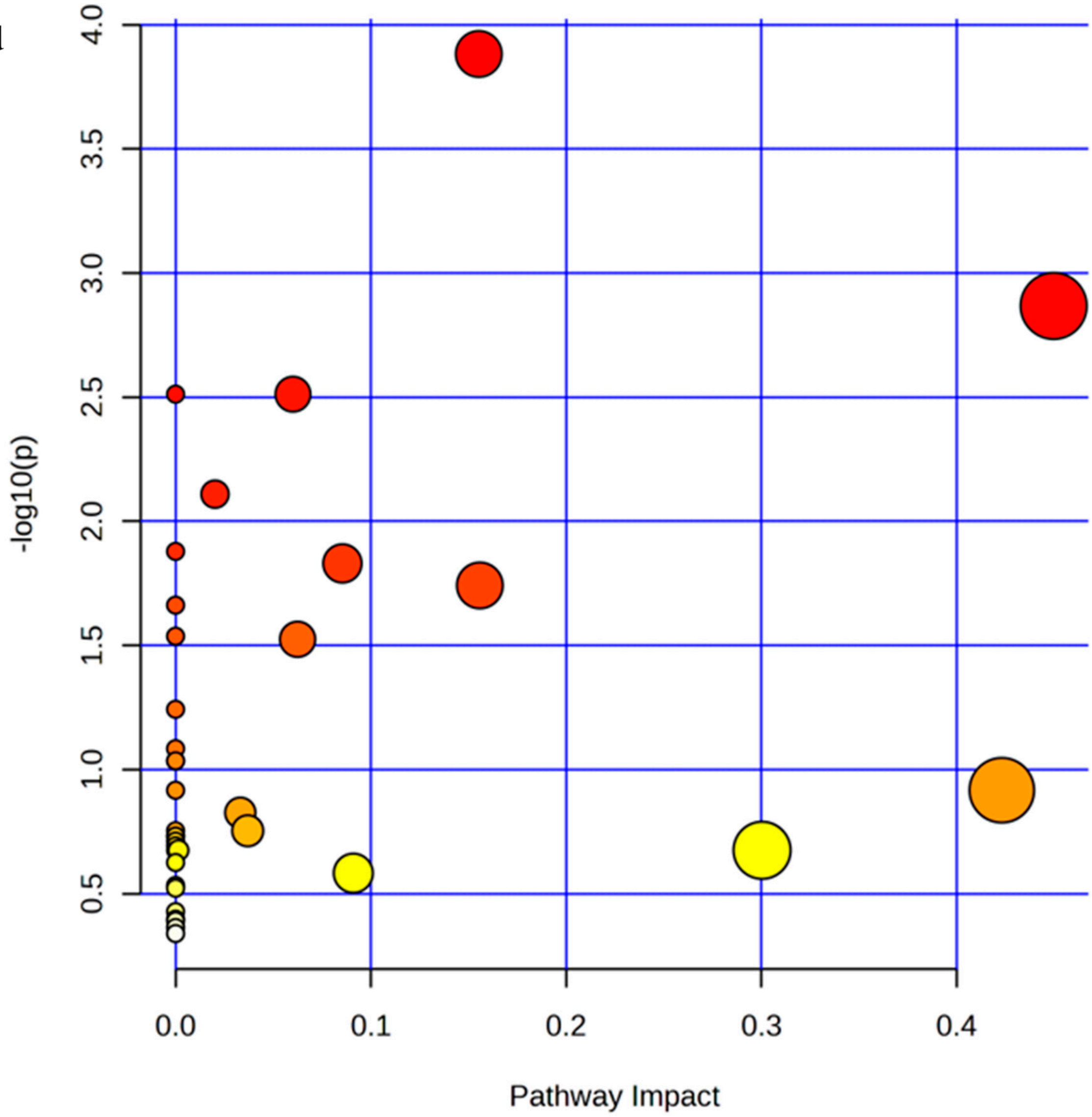


Figure. S3 Metabolic pathways of P20 (a, b), P20V10 (c, d) compared with CK. Up (a and c), down (b and d)
